# ZEB1 turns into a transcriptional activator by interacting with YAP1 in aggressive cancer types

**DOI:** 10.1038/ncomms10498

**Published:** 2016-02-15

**Authors:** Waltraut Lehmann, Dirk Mossmann, Julia Kleemann, Kerstin Mock, Chris Meisinger, Tilman Brummer, Ricarda Herr, Simone Brabletz, Marc P. Stemmler, Thomas Brabletz

**Affiliations:** 1 Department of General and Visceral Surgery, University of Freiburg Medical Center, Hugstetter Strasse 55, 79106 Freiburg, Germany; 2 Faculty of Biology, Albert-Ludwigs-University Freiburg, 79104 Freiburg, Germany; 3 Institute for Biochemistry and Molecular Biology, ZMBZ, Albert-Ludwigs-University Freiburg, Stefan-Meier-Strasse 17, 91054 Freiburg, Germany; 4 Department of Experimental Medicine 1, Nikolaus-Fiebiger-Center for Molecular Medicine, FAU University Erlangen-Nürnberg, Glückstrasse 6, 91054 Erlangen, Germany; 5 BIOSS Centre for Biological Signalling Studies, Albert-Ludwigs-University Freiburg, 79104 Freiburg, Germany; 6 Institute for Molecular Medicine and Cell Research, Albert-Ludwigs-University Freiburg, Stefan-Meier-Strasse 17, 91054 Freiburg, Germany

## Abstract

Early dissemination, metastasis and therapy resistance are central hallmarks of aggressive cancer types and the leading cause of cancer-associated deaths. The EMT-inducing transcriptional repressor ZEB1 is a crucial stimulator of these processes, particularly by coupling the activation of cellular motility with stemness and survival properties. ZEB1 expression is associated with aggressive behaviour in many tumour types, but the potent effects cannot be solely explained by its proven function as a transcriptional repressor of epithelial genes. Here we describe a direct interaction of ZEB1 with the Hippo pathway effector YAP, but notably not with its paralogue TAZ. In consequence, ZEB1 switches its function to a transcriptional co-activator of a ‘common ZEB1/YAP target gene set', thereby linking two pathways with similar cancer promoting effects. This gene set is a predictor of poor survival, therapy resistance and increased metastatic risk in breast cancer, indicating the clinical relevance of our findings.

## Abstract (web summary)

The transcription factors ZEB1 and YAP function in different pathways yet both activate aggressive behaviour in cancer cells. Here, the authors describe that the proteins physically interact and that this changes the transcriptional activity of ZEB1 from a repressor to an activator.

## Graphical abstract

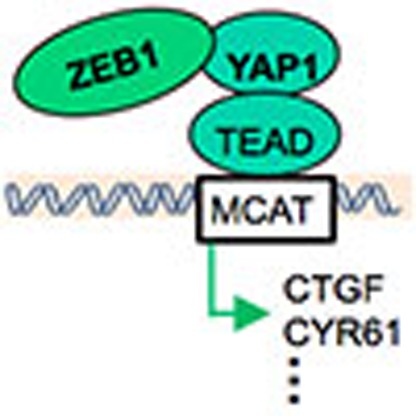

Central hallmarks of cancer progression and aggressiveness are tumorigenic capacity, dissemination, metastasis and resistance to conventional radio/chemotherapy. These traits are responsible for the major clinical problems and prevent successful treatment of many cancer patients. The uncovering of the underlying molecular mechanisms is crucial for developing novel therapeutic concepts.

In the past decade it became evident that, like in leukemia, also in most solid cancers, a subpopulation of tumour cells termed cancer stem cells (CSCs) has high tumour-initiating capacity and is the source of metastasis and treatment relapse^1^,^2^. Notably, it has been demonstrated that the embryonic epithelial–mesenchymal transition (EMT)-program can be activated in cancer cells, which not only induces an aberrant motility triggering dissemination and metastasis, but also confers stemness properties resulting in a migrating CSC-phenotype^3^,^4^. The program is activated by EMT-inducing transcription factors including members of the Snail-, Twist- and ZEB families^5^.

The EMT-activator ZEB1 turned out to be particularly potent^6^,^7^. ZEB1 is associated with aggressive behaviour, metastasis, treatment resistance and poor prognosis in different tumour types, including breast, pancreatic and lung cancer^8^,^9^,^10^. In breast cancer, highest ZEB1 expression in tumor cells was found in the aggressive triple negative and basal types^9^,^11^ and to be upregulated in circulating tumour cells with a CSC-phenotype^12^. Mechanistically, ZEB1 is a transcriptional repressor of epithelial genes, for example, for E-cadherin and cell polarity factors, thereby stimulating an undifferentiated and highly motile phenotype^13^. This property of ZEB1 is considered important for metastasis as shown in many model systems^10^,^14^,^15^,^16^,^17^. By repressing the expression of the stemness-inhibiting microRNAs miR-200 and miR-203, ZEB1 can also confer stemness properties, thereby linking motility and stemness towards a migrating cancer stem cell phenotype^17^,^18^. Moreover ZEB1, likely through its stemness-promoting effect, can confer survival and therapy resistance, as shown for many different cancer types, such as pancreatic, breast and lung cancer^17^,^19^,^20^,^21^. Owing to these pleiotropic effects, ZEB1 is considered the central factor for high cancer cell plasticity as a motor towards aggressive, metastatic and therapy-resistant cancer types^22^,^23^.

However, the strong effects of ZEB1 cannot be solely explained by the ascribed functions as a transcriptional repressor. In this study, by analysing ZEB1-dependent gene expression patterns, we demonstrate mechanistic links explaining the extraordinary potency of ZEB1 in driving tumour progression. We describe a direct interaction between ZEB1 and the Hippo pathway effector YAP, shifting ZEB1 from a repressor to a transcriptional activator and thereby linking two pathways with very similar cancer-promoting effects. Notably, ZEB1 directly binds to YAP, but not to the paralogue factor TAZ. Functional cooperation of ZEB1 and YAP stimulates the transcriptional activation of a ‘common ZEB1/YAP target gene set'. This gene set is a predictor of poor survival, therapy resistance and increased metastatic risk in hormone receptor-negative breast cancer, indicating the clinical relevance of our findings.

## Results

### YAP target gene expression depends on ZEB1

**Figure 1:**
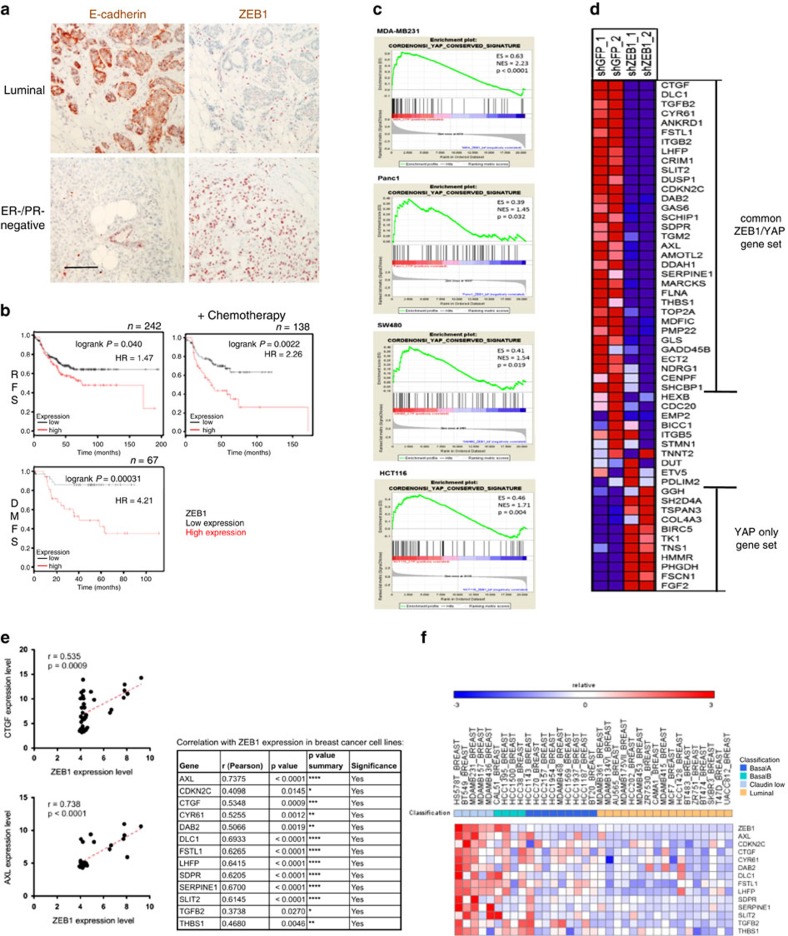
ZEB1 correlates with YAP target gene expression. (**a**) Representative immunohistochemistry of different breast cancers for E-cadherin and ZEB1, showing mutual exclusion of both proteins in cancer cells. Scale bars, 40 μm. (**b**) Kaplan–Meier plots from meta-analyses showing relapse-free survival (RFS), relapse-free survival after adjuvant chemotherapy and distant metastasis-free survival (DMFS) of ER^−^/PR^−^ breast cancers based on ZEB1 expression. Log-rank test. (**c**) Gene set enrichment analysis (GSEA) of microarray data sets from cancer cell lines of different entities reveals enrichment of conserved YAP target genes in MDA-MB231 (breast), HCT116 (colon), SW480 (colon) and Panc1 (pancreas) control cells compared with shZEB1 knockdown. ES, enrichment score; NES, normalized enrichment score. (**d**) Heat map depicting the differential expression of YAP signature genes in two MDA-MB231 control (shGFP) compared with two stable ZEB1 knockdown clones (shZEB1, ZEB1 knockdown to 4.4 and 7.2% of shCTR). Red and blue indicate high and low mRNA expression levels, respectively. (**e**) Significant correlations between mRNA expression of ZEB1 and YAP target genes in breast cancer cell lines from the cancer cell line encyclopedia (CCLE). Pearsons correlation coefficient. (**f**) Heat map showing high expression levels of ZEB1 and YAP target genes in aggressive breast cancer cell lines (GSE36133), predominantly in claudin-low and Basal B subtypes.

ZEB1 expression in tumour cells of human cancers is heterogenous. In breast cancer, the aggressive triple-negative (ER−, PR−, HER2−) or the basal subtypes often express high amounts (Fig.1a). ZEB1 expression in these subtypes is correlated with poor survival, therapy resistance and high risk for distant metastasis (Fig. 1b). We wanted to further investigate ZEB1-dependent mechanisms resulting in aggressive cancer types. Gene expression analyses comparing aggressive cancer cells with high ZEB1 levels of different entities (breast, pancreas, colon) and corresponding ZEB1 knockdown cells revealed a strong reprogramming with expression changes (>3-fold up or down) in thousands of genes. About 60% of the changes resulted from genes which were upregulated upon knockdown of ZEB1 and thus represent potential direct targets of its well-established transcriptional repressor function. However, the remaining 40% of the genes were downregulated upon knockdown, indicating that their expression directly or indirectly (for example, through upregulation of miR-200) depends on the aberrant expression of ZEB1 in cancer cells. Accordingly, we hypothesized that for certain gene patterns, ZEB1 can switch from a transcriptional repressor to a transcriptional activator.

Gene set enrichment analysis (GSEA) of ZEB1-dependent genes in breast, colon and pancreatic cancer cells revealed gene sets strongly enriched compared with ZEB1 knockdown cells (Supplementary Fig. 1a). One of the most significant overlaps regarding all analysed cancer cell entities was found for an evolutionary conserved signature of the Hippo-pathway effector YAP (Yes-associated protein) described by Cordenonsi *et al.*^24^ (Fig. 1c and Supplementary Fig. 1b). This strong overlap is striking, since the Hippo-pathway and particularly its main downstream effectors, the transcriptional co-activators YAP and TAZ (transcriptional co-activator with PDZ-binding motif), have emerged as having important roles during tumour formation and malignant progression^25^,^26^. There is a remarkable congruency in the effects of YAP/TAZ with those of ZEB1. As demonstrated for ZEB1, also YAP/TAZ expression is associated with aggressive behaviour, metastasis, treatment resistance and poor prognosis in different tumour types^27^,^28^,^29^. Also YAP/TAZ can confer stemness traits, therapy resistance and metastasis in experimental models of different tumour types including mammary tumours^24^,^30^. We focused on breast cancer and particularly on those genes of the YAP target gene set that were most differentially regulated between control and ZEB1 knockdown cells and thus contributed most to the enrichment result. In the triple-negative breast cancer line MDA-MB231, ZEB1 is aberrantly expressed^11^ and, as also described for YAP^31^, is important for the invasive, metastatic, drug-resistant and stemness phenotype of this cell line^16^,^32^. In MDA-MB231, most of the described YAP target genes were also depending on the expression of ZEB1 (termed ‘common ZEB1/YAP set') (Fig. 1d). About 20% of YAP target genes were not downregulated, but upregulated upon ZEB1-knockdown (termed ‘YAP only gene set'). We further extended this finding by analysing breast cancer cell lines from the cancer cell line encyclopedia^33^, which revealed strong correlations of ZEB1 and common ZEB1/YAP target gene expression (Fig. 1e and Supplementary Fig. 1c). Gene expression analyses in breast cancer have led to the definition of five molecular subtypes (luminal A, luminal B, HER2-enriched, basal-like and claudin-low) and the claudin-low subtype has been characterized as a subtype of aggressive triple-negative breast cancers displaying a high enrichment for EMT-markers (including ZEB1) and genes providing stem cell-like features^34^. Genes of the common ZEB1/YAP target gene set were highly expressed in breast cancer cell lines assigned to the claudin-low and basal B subtype (Fig. 1f).

**Figure 2:**
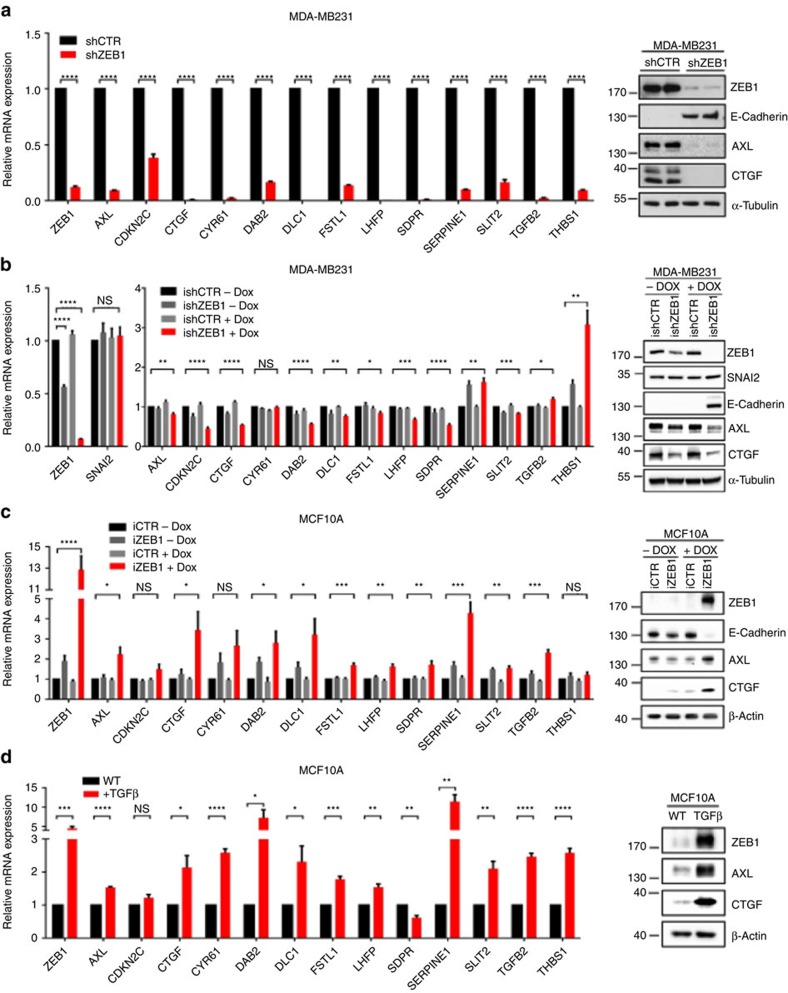
ZEB1 is critical for YAP target gene expression. (**a**) Stable knockdown of ZEB1 in MDA-MB231 cells results in downregulation of YAP target genes on mRNA and protein level, analysed by qRT–PCR and western blot; *n*=3. (**b**) qRT–PCR and western blot analysis of ZEB1, SNAI2 and YAP target genes in MDA-MB231 cells. ZEB1 knockdown was induced by the addition of doxycycline for 6 days; *n*=3. (**c**) qRT–PCR and western blot analysis of ZEB1 and YAP target genes in MCF10A cells with or without doxycycline-induced ZEB1 expression; *n*=4. (**d**) TGFβ induces expression of ZEB1 and YAP target genes in MCF10A cells, qRT–PCR and western blot; *n*=4. For **a**–**d**, mean±s.e.m. *P=0.01–0.05, **P=0.001–0.01, ***P=0.001–0.0001, ****P<0.0001, NS, not significant, unpaired two-tailed Student's *t*-test.

We validated the findings on mRNA level and further showed that ZEB1 depletion in MDA-MB231 reduced protein expression of the YAP/TAZ targets *CTGF* and *AXL*. This was true for cells carrying a stable ZEB1 knockdown (Fig. 2a), and for a doxycycline (dox)-inducible ZEB1-depletion (Fig. 2b), thus excluding that the observations were due to selective or adaptive effects of long-term ZEB1 depletion. Vice versa, dox-induced overexpression of ZEB1 in the human mammary epithelial cell line MCF10A, expressing low levels of ZEB1, led to upregulation of YAP target genes (Fig. 2c). As expected, expression of ZEB1 resulted in an EMT, indicated by typical morphological changes and downregulation of the epithelial marker E-cadherin (Supplementary Fig. 2a). These findings suggest that in addition to its well-characterized role as transcriptional repressor of epithelial genes, ZEB1 co-activates the expression of a selective YAP-target gene set. We further used another, more physiological setting of ZEB1 induction by treating MCF10A cells with TGFβ until the cells had entered a stable EMT state as judged by cell morphology and ZEB1 expression. In line with the results of ectopically expressed ZEB1, TGFβ-induced ZEB1 expression also activated the expression of YAP target genes (Fig. 2d and Supplementary Fig. 2a).

Here, we describe a new subset of genes depending on ZEB1 expression, which strongly overlaps with a YAP target gene signature. The importance of YAP/TAZ target genes such as *CTGF*, *CYR61*, *AXL* and others for cancer progression including metastasis^35^, drug resistance^36^,^37^ and poor clinical outcome^38^ was already demonstrated. Thus the detection of a common ZEB1/YAP target gene set points to a new mechanism how ZEB1 drives malignant cancer progression, towards invasion, metastasis and therapy resistance.

### The regulation of YAP target genes is ZEB1 specific

**Figure 3:**
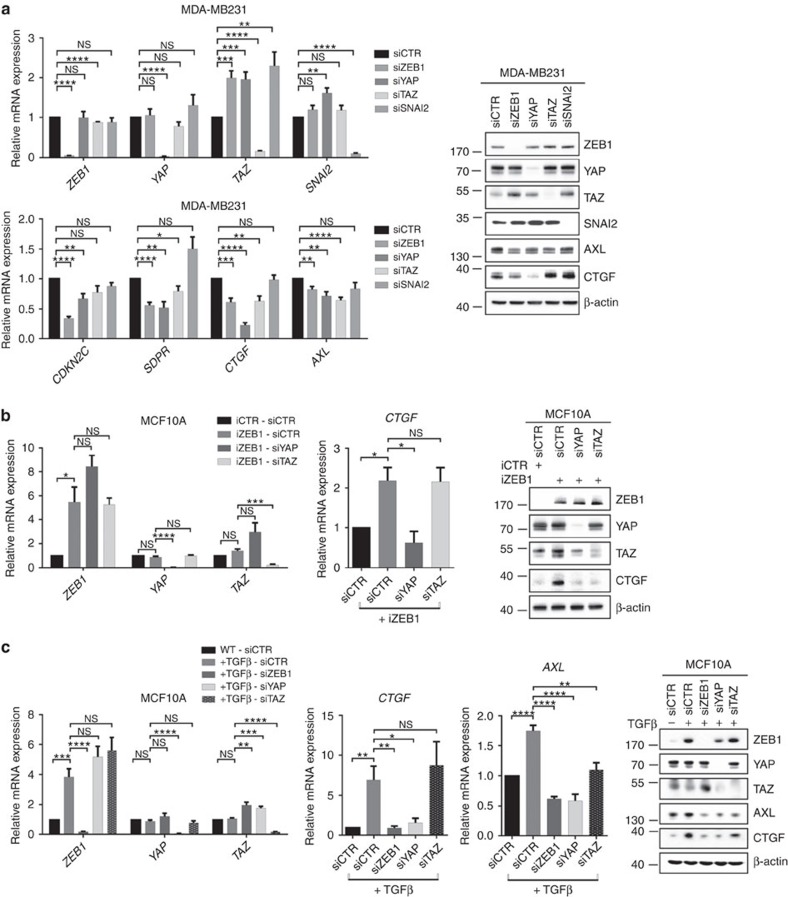
Activation of YAP target genes is ZEB1 specific and requires YAP. (**a**) Expression of indicated genes in MDA-MB231 cells upon transient knockdown with the indicated siRNAs. Reduced expression of indicated YAP target genes upon siRNA-mediated knockdown of ZEB1, YAP or TAZ but not SNAI2 in MDA-MB231 cells. mRNA and protein expression were analysed by qRT–PCR and western blot; *n*=5. (**b**) qRT–PCR and western blot analysis of MCF10A cells. The iCTR and iZEB1 cells were treated with doxycycline for 6 days and siRNA-mediated knockdown of YAP or TAZ was performed after 3 days for 72 h; *n*=4. (**c**) Depletion of ZEB1, YAP or TAZ reverts TGFβ-induced expression of YAP target genes in MCF10A cells. siRNA-mediated knockdown in WT or TGFβ-treated MCF10 cells was performed for 72 h and cells were analysed by qRT–PCR or western blot; *n*=6. For **a**–**c**, mean±s.e.m. *P=0.01–0.05, **P=0.001–0.01, ***P=0.001–0.0001, ****P<0.0001, NS, not significant, unpaired two-tailed Student's *t*-test.

We further determined the interdependence of ZEB1, the Hippo effectors YAP/TAZ and the EMT-activator SNAI2 (Slug), which is co-expressed with ZEB1 in the investigated cell lines. In line with results for stable ZEB1 depletion, transient ZEB1 knockdown significantly decreased the expression of YAP target genes to an extent comparable with YAP knockdown (Fig. 3a). Notably, loss of ZEB1 did not interfere with YAP expression and even slightly upregulated TAZ. In contrast, knockdown of the EMT-activator SNAI2 did not affect YAP target gene expression. This is further in line with the finding that ZEB1 knockdown in MDA-MB231, although downregulating YAP target genes, did not affect expression of SNAI2 (Fig. 2b), ruling out that the effects of ZEB1 depletion were mediated indirectly by reduction of SNAI2 and that SNAI2 can substitute for a loss of ZEB1 in this context. To further determine whether ZEB1-mediated activation of YAP targets is depending on YAP or TAZ, we depleted both factors in ZEB1-overexpressing MCF10A cells (Fig. 3b). Knockdown of YAP reverted the ZEB1-induced expression of *CTGF* without affecting ZEB1 levels, indicating that YAP is necessary for this function of ZEB1. Moreover, depletion of TGFβ-induced ZEB1 in MCF10A cells was sufficient to abrogate the expression of YAP target genes to an extent comparable to YAP knockdown (Fig. 3c and Supplementary Fig. 2b). Depletion of TAZ had no consistent effect in this setting. These data further support a role of ZEB1 and YAP in activating a common target gene set important for tumour progression.

### ZEB1 does not modulate YAP phosphorylation or localization

**Figure 4:**
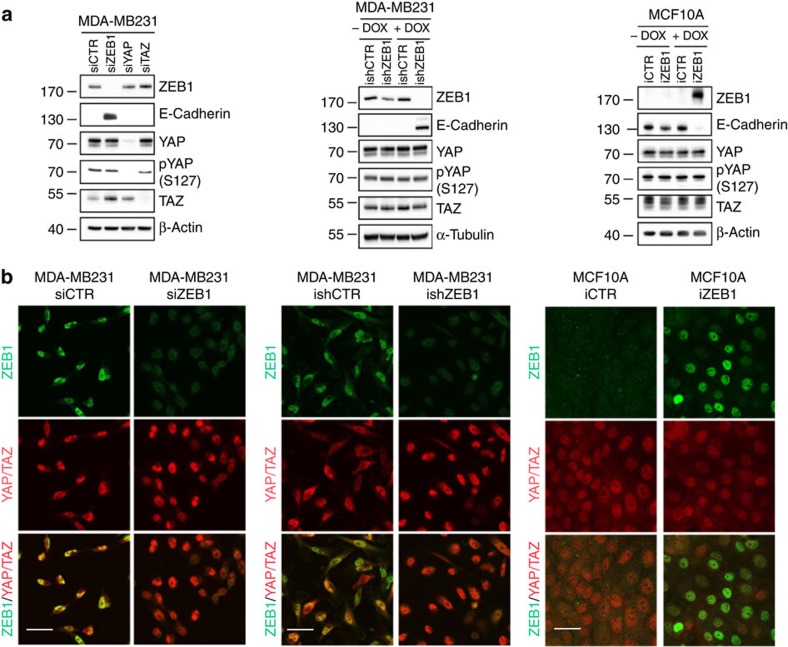
ZEB1 does not affect YAP phosphorylation or localization. (**a**) Western blot analysis of MDA-MB231 cells 72 h after siRNA-mediated knockdown of ZEB1, YAP or TAZ, of MDA-MB231 cells 6 days after induction of ZEB1 knockdown by doxycycline or of MCF10A cells 6 days after induction of ZEB1 expression by doxycycline. Phosphorylation of the LATS kinase target residue serine 127 (S127) of YAP was assessed with a pS127- specific antibody. (**b**) Immunofluorescence staining of MDA-MB231 cells 72 h after siRNA-mediated knockdown of ZEB1, MDA-MB231 cells 6 days after induction of ZEB1 knockdown by doxycycline or of confluent MCF10A cells 3 days after induction of ZEB1 expression by doxycycline. Scale bars, 20 μm.

Upstream regulators of the Hippo pathway include cell polarity factors such as the apical Crumbs and the basal Scribble complex^24^,^39^ and EMT-triggered delocalization or loss of Scribble results in reduced LATS-mediated phosphorylation of YAP/TAZ subsequently increasing protein stability^24^. We have previously shown that ZEB1 directly represses expression of cell polarity factors including members of the *Crumbs* and *Scribble* family, resulting in a loss of cell polarity^16^,^40^. We therefore wanted to rule out that ZEB1 indirectly stimulates YAP target gene expression by preventing Crumbs- or Scribble-dependent YAP/TAZ degradation. First, we assayed YAP and TAZ protein levels by western blot analysis and did not observe any reduction in response to ZEB1-knockdown or increase after overexpression of ZEB1, respectively. Moreover, also phosphorylation of YAP on serine 127, a target site of LATS kinase downstream of the Hippo pathway^41^, was not altered (Fig. 4a). Second, we monitored the subcellular localization of YAP/TAZ, which is nuclear in their dephosphorylated, active state but re-localizes to the cytoplasm upon activation of LATS-mediated phosphorylation^42^. In MDA-MB231 cells siRNA-mediated or Dox-induced depletion of ZEB1 had no effect on YAP/TAZ nuclear localization (Fig. 4b). Also in MCF10A cells, YAP/TAZ localization was irrespective of the presence or absence of ZEB1. Hippo-pathway effectors were shown to activate EMT^43^,^44^. Particularly, TAZ was shown to activate ZEB1 expression in retinal pigment epithelial cells^45^. Therefore, we tested whether YAP and TAZ can stimulate ZEB1 in our cellular systems. However, neither depletion of YAP or TAZ, nor overexpression of both factors affected expression and nuclear localization of ZEB1 (Figs 3a,c and 4a and Supplementary Fig. 2c, d). In conclusion, regulation of YAP target genes by ZEB1 cannot simply be ascribed to a Hippo-pathway mediated regulation of YAP/TAZ activity, for example, through ZEB1-induced loss of cell polarity.

### ZEB1 functionally interacts with YAP

**Figure 5:**
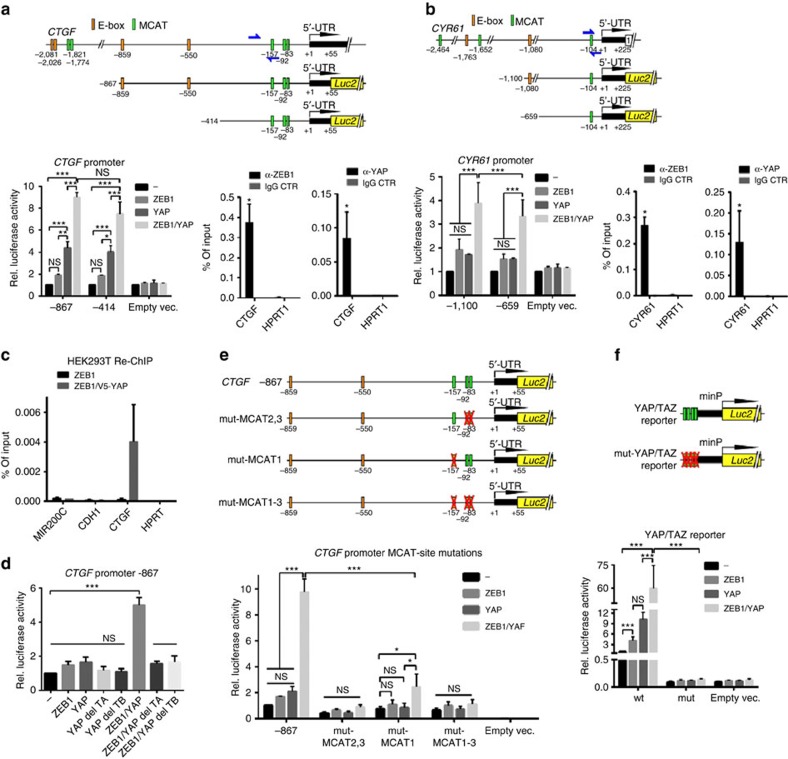
ZEB1 functionally interacts with YAP. (**a**,**b**) Schematic representation of the promoter region of human *CTGF* (**a**) and *CYR61* (**b**) genes on chromosomes 6q23.2 and 1p22.3, respectively. The potential ZEB1 (E-boxes) and YAP/ TEAD (MCAT) binding sites, the regions amplified after chromatin immunoprecipitation (ChIP) (blue arrows represent primer pairs) are depicted. Numbers indicate positions in bp on chromosomal DNA relative to the transcription start site (+1). Used luciferase (*luc2*) reporter constructs with and without E-boxes analysed in MCF7 cells transiently transfected with ZEB1, YAP or empty vector control are shown; *n*=3. qPCR analysis after ChIPs for endogenously expressed ZEB1 and YAP in MDA-MB231 cells shows direct binding of both factors, the promoters of *CTGF* and *CYR61*. *HPRT1* is used as negative control; *n*=3. (**c**) qPCR analyses of anti-ZEB1/anti-YAP sequential ChIP (re-ChIP) of HEK293T cells overexpressing ZEB1 alone or in combination with V5-tagged YAP at the indicated gene loci (initial ChIP against ZEB1 and re-ChIP against V5). We confirmed that ZEB1 and YAP can simultaneously bind to the same region of the *CTGF* gene but not at the *CDH1* and *MIR200C* loci, two genes repressed by ZEB1 and at control *HPRT1*; *n*=2. (**d**) CTGF-promoter reporter assay upon overexpression of ZEB1 and YAP expression constructs (YAP=wild type, del TA=without transactivation domain, del TB=without TEAD binding domain) in MCF7; *n*=3. (**e**) *CTGF*-promoter reporter constructs with sequential mutations in the three TEAD bindings sites (red crosses). Reporter assays in MCF7 cells showing that TEAD binding sites are important for functional interaction of ZEB1 and YAP; *n*=3. **(f)** Reporter constructs with four tandem repeats of wild-type or mutated (mut) TEAD binding sites upstream of a minimal promoter analysed in MCF7 cells showing that functional interaction of ZEB1 and YAP is transferred through TEAD binding sites *n*=3. For **a**–**f**, mean±s.e.m. For reporter assays, firefly luciferase activity was normalized to co-transfected *renilla* luciferase; *P=0.01–0.05, **P=0.001–0.01, ***P=0.001–0.0001, ****P<0.0001, NS, not significant, two-way analysis of variance with Bonferroni multiple comparisons test. ****P<0.0001, unpaired two-tailed Student's *t*-test. For ChIP assays, *P=0.01–0.05, nonparametric Mann–Whitney *U*-test.

YAP/TAZ and ZEB1 co-localized in the nucleus (Fig. 4b) and ZEB1 directly binds to DNA by two zinc-finger clusters for recognizing so called E-boxes^13^. YAP/TAZ have no direct DNA-binding capacity but need to interact with members of the TEAD family, important mediators of the oncogenic properties of YAP/TAZ^25^,^29^. Binding of YAP to the promoters of *CTGF and AXL* was previously shown^46^,^47^. We performed chromatin-immunoprecipitation (ChIP) analyses and demonstrated that ZEB1 and YAP bind to the *CTGF, CYR61, SDPR* and *AXL* promoters (Fig. 5a,b and Supplementary Fig. 3a). By applying a sequential ChIP (first ChIP for ZEB1 and re-ChIP for V5-tagged YAP), we confirmed that ZEB1 and YAP can simultaneously bind to the same promoter region at the *CTGF* locus in a native context (Fig. 5c and Supplementary Fig. 3b). Of note, only ZEB1 but not simultaneous binding of YAP was detected at the *CDH1* and *MIR200C* loci, two genes which are repressed by ZEB1. We further confirmed *CTGF* as direct ZEB1 target by demonstrating that ZEB1 synergizes with YAP in stimulating *CTGF* and *CYR61* promoter activity. Unexpectedly, E-boxes as known ZEB1 binding sites are dispensable for functional interaction of ZEB1 and YAP, since deletion of E-box containing promoter regions did not significantly reduce the co-stimulation (Fig. 5a,b). Deletion of either the TEAD binding domain or the transactivation domain of YAP completely abolished the co-activation by YAP and ZEB1 (Fig. 5d). These results indicated that not the known ZEB1 binding sites (E-boxes) but the YAP/TEAD bindings sites (MCATs) are crucial for the functional interaction of ZEB1 and YAP in stimulating transcription. This assumption was further fostered by showing that sequential mutation of the TEAD binding sites inhibited co-stimulation of the *CTGF* promoter by ZEB1 and YAP (Fig. 5e). Finally, we cloned a polymerized TEAD binding site upstream of a minimal promoter, and demonstrated that ZEB1 and YAP can strongly co-stimulate the reporter gene in a highly synergistic manner, whereas mutation of the TEAD sites abolished this effect (Fig. 5f). Our findings demonstrate that ZEB1 and YAP can functionally interact to co-activate common target genes through conserved TEAD binding sites. Binding of ZEB1 to E-boxes, its known binding elements, does not seem to be mandatory for this function of ZEB1.

### ZEB1 directly binds to YAP but not to TAZ

**Figure 6:**
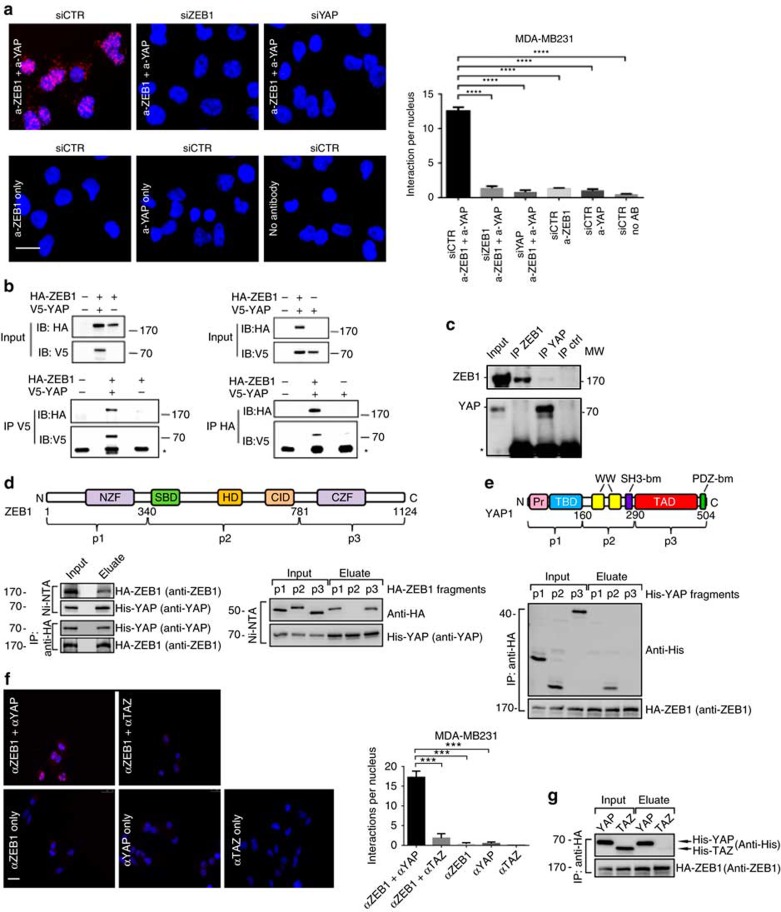
ZEB1 directly binds to YAP but not to TAZ. (**a**) *In situ* proximity ligation assay (PLA) shows an interaction between ZEB1 and YAP in the nucleus of MDA-MB231 cells indicated by red fluorescent spots. Transient siRNA-mediated knockdown of ZEB1 or YAP or incubation without antibody or either ZEB1 or YAP antibody alone were used as controls. Representative microscopic images are shown and five images from each condition were quantified, counting the number of interactions per nucleus; *n*=3. (**b**) Co-immunoprecipitation (CoIP) of ectopically expressed HA-tagged ZEB1 and V5-tagged YAP from nuclear extracts of MCF7 cells reveals an interaction between ZEB1 and YAP proteins on western blot. IP, immunoprecipitation; IB, immunoblot. (**c**) CoIP of endogenous ZEB1 and YAP in MDA-MB231 cells showing co-precipitated YAP after ZEB1 immunoprecipitation. (**d**) Schematic representation of the *in vitro* synthesized HA-tagged ZEB1 fragments p1–3 (numbers are amino acids). p1, 2 and 3 correspond to the N-terminal zinc finger cluster, the central part and the C-terminal zinc finger cluster of ZEB1, respectively. *In vitro* pull-down assay of full-length HA-ZEB1 by anti-HA affinity matrix and full-length His-YAP by Ni-NTA shows co-precipitation of His-YAP and HA-ZEB1, respectively (left). *In vitro* pull-down assay of full-length His-YAP by Ni-NTA and co-precipitation of the indicated ZEB1 fragments (right). Note that YAP binds to the regions containing the zinc-finger clusters, but not the central parts of ZEB1. (**e**) Identical strategy for YAP (His-tagged) as followed for ZEB1 in **d**, showing that YAP interacts with ZEB1 specifically through its central WW-repeat containing domain p2. (**f**) PLA assay showing an interaction of ZEB1 with YAP but not with TAZ in nuclei of MDA-MB231; *n*=3. (**g**) *In vitro* pull-down assay of full-length HA-ZEB1 by anti-HA affinity matrix and western blot (anti-His) for full-length His-YAP or His-TAZ, demonstrating that ZEB1 binds to YAP but not to TAZ. For **a** and **f**, mean±s.e.m.; ***P=0.001–0.0001, ****P<0.0001, unpaired two-tailed Student's *t*-test. Scale bar, 10 μm.

We next investigated, whether ZEB1 and YAP can also directly interact. *In situ* proximity ligation assay visualized endogenous ZEB1–YAP protein complexes in the nucleus of MDA-MB231 or ZEB1-overexpressing MCF10A cells (Fig. 6a and Supplementary Fig. 4a). Co-immunoprecipitation (CoIP) of ectopically expressed HA-tagged ZEB1 or V5-tagged YAP from nuclear extracts confirmed the interaction between ZEB1 and YAP (Fig. 6b and Supplementary Fig. 4b), which was also shown for endogenous ZEB1 and YAP, after IP of YAP (Fig. 6c). Using *in vitro* translated HA-tagged ZEB1 and His-tagged YAP, we identified that the two proteins directly interact by anti HA- and His-pull-downs in both directions (Fig. 6d). By using *in vitro* translated non-overlapping sub-fragments of both factors we could further narrow down the interaction domains. YAP binds to the amino (N)- and carboxy (C)-terminal domains of ZEB1 including the two zinc-finger clusters (Fig. 6d) and ZEB1 binds to central regions of YAP containing the WW-domains (Fig. 6e). We next wanted to know whether the interaction is specific for YAP, or if ZEB1 is also binding to TAZ, the paralogue of YAP with redundant, undistinguishable function. Notably, an interaction of ZEB1 with TAZ was not detected in the proximity ligation assay (Fig. 6f), although both factors were strongly expressed in the nuclei (Supplementary Fig. 4c). Also CoIP assays of purified, tagged ZEB1 and TAZ proteins revealed no direct binding to each other (Fig. 6g). Thus, the ZEB1/YAP interaction seems to be highly specific, which indicates a functional difference between YAP and TAZ.

### Clinical relevance of common ZEB1/YAP targets

**Figure 7:**
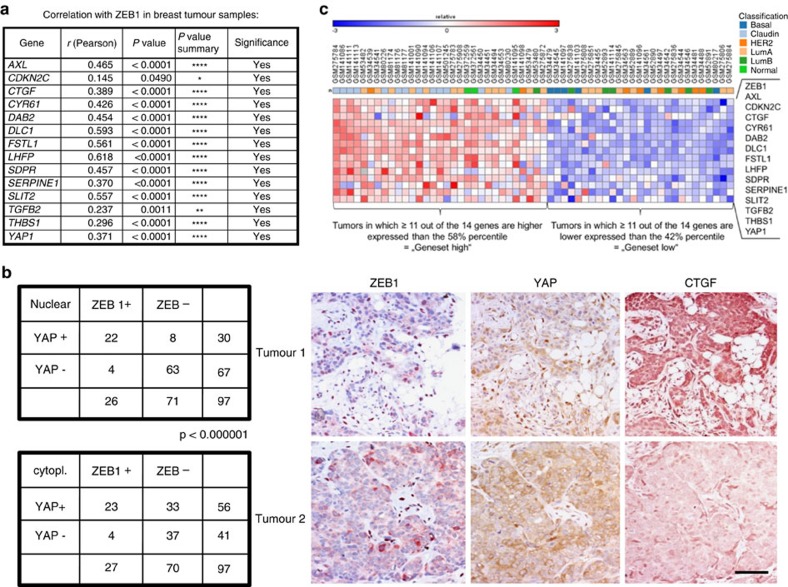
Correlated expression of ZEB1 and YAP with their target genes. (**a**) Significant correlation between mRNA expression of ZEB1, YAP and ZEB1/YAP target genes in 186 breast cancer patient samples (GSE18229). Pearsons correlation coefficient. (**b**) Significant correlation of ZEB1 and YAP expression as well as localization (nuclear versus cytoplasmic) on protein level as determined by immunohistochemistry of 97 human breast cancers. The right panel shows a representative serial section of breast cancers stained for ZEB1 and YAP, demonstrating correlated nuclear (tumour 1) or cytoplasmic (cytopl.; tumour 2) expression of both proteins in cancer cells. CTGF is strongly expressed in tumour 1 showing nuclear ZEB1 and YAP expression. Fisher's exact test; Scale bars, 40 μm. (**c**) Heat map showing correlated expression of ZEB1, YAP and ZEB1/YAP target genes in breast cancer patient samples (GSE18229) defined as ‘gene set high' compared with tumours defined as ‘gene set low' and the corresponding subtype classification of these tumours.

**Figure 8:**
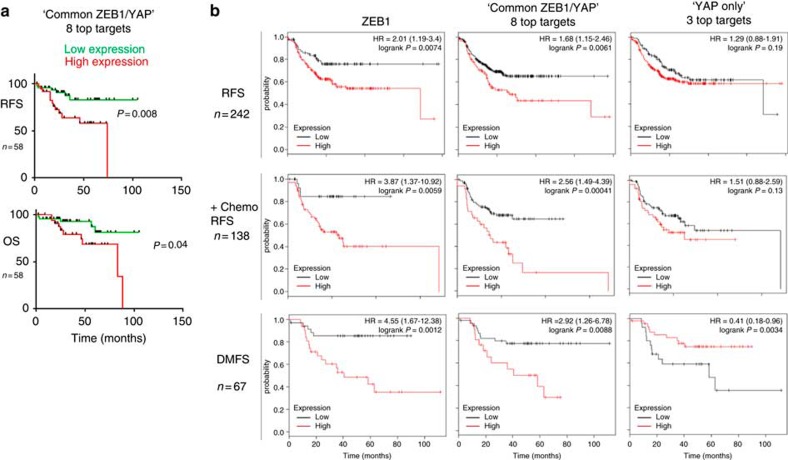
Clinical relevance of common ZEB1/YAP target genes. (**a**) Kaplan–Meier plots representing the probability of cumulative relapse-free (RFS) or overall survival (OS) in the same breast cancer set as in **b** (GSE18229) stratified according to the expression status of the top 8 genes of the ‘common ZEB1/YAP target gene set' (including AXL, LHFP, SERPINE1, SLIT2, DAB2, FSTL1, THBS1, CTGF) in their primary tumours. The log-rank test *P* value reflects the significance of the correlation between gene set high and shorter survival outcome. (**b**) Kaplan–Meier plots from meta-analyses showing relapse-free survival (RFS), relapse-free survival after adjuvant chemotherapy and distant metastasis-free survival (DMFS) of ER^−^/PR^−^ breast cancers based on expression of the indicated genes. Note the inverse behaviour of the ‘common ZEB1/YAP top 8 target set' and the ‘YAP only top 3 target set‘ (including HMMR, BIRC5, GGH), particularly for DMFS. For Kaplan–Meier plots, the log-rank test was applied.

We further validated the translational and clinical relevance of a functional ZEB1/YAP interaction and its common target gene activation by analysing human breast cancer data sets. The mRNA expression of ZEB1, YAP and genes of the common ZEB1/YAP target set is significantly correlated in human breast cancers (Fig. 7a). Since ZEB1 is also expressed in tumour stromal cells, we analysed expression in cancer cells by immunohistochemistry of human breast cancers and also considered protein localization. Nuclear expression of ZEB1 was significantly correlated with nuclear expression of YAP in breast cancer (Fig. 7b). In cases with exclusive cytoplasmic ZEB1 expression, we also detected a correlation with cytoplasmic YAP expression. As detected in breast cancer cell lines (Fig. 1f), molecular subtype analyses of human breast tumours revealed a high expression of ZEB1 and common ZEB1/YAP target genes in the claudin-low type, but also in a fraction of other subtypes, including Her2+, normal-like and luminal A types. Irrespective of the subtype, we again detected a correlated expression of ZEB1, YAP and common ZEB1/YAP target genes (Fig. 7c). Moreover, survival analysis of this breast cancer data set demonstrated that irrespective of the subtype, tumours with high expression of the ‘common ZEB1/YAP target genes' displayed a significant shorter relapse-free and overall survival (Fig. 8a). The clinical relevance of the common ZEB1/YAP target gene set could be further validated by performing a meta-analysis for breast cancers conventionally classified as hormone receptor negative (ER−/PR−), which include the most aggressive subtypes^48^. As shown for ZEB1 (Fig. 1b), individual members of the ‘common ZEB1/YAP target gene set', as well as a cluster of the top eight genes strongly correlated with poor relapse-free survival, resistance to chemotherapy and high risk for distant metastasis (Fig. 8b and Supplementary Fig. 5a). These results demonstrate a high tumour biological and clinical relevance of a functional interaction between ZEB1 and YAP. As a control, we analysed genes of the ‘YAP only' target gene set, which were even downregulated in the presence of ZEB1 (‘YAP only set', see Fig. 1d). Strikingly, expression of individual members of the ‘YAP only set' as well as a cluster of the top three genes of this set did not correlate with poor prognosis, but even indicated better therapy response and particularly reduced metastatic risk (Fig. 8b and Supplementary Fig. 5b).

## Discussion

Here, we describe a direct functional link between ZEB1 and the transcriptional co-activator YAP. Mechanistically, we found that ZEB1, in a complex with YAP, binds to gene promoters resulting in transcriptional activation of YAP target gene expression. Analysis of clinical data and patient material provides evidence for a particular relevance of our findings in aggressive breast cancer types, showing that a ‘common ZEB1/YAP target gene set' is a strong predictor of poor relapse free survival, therapy resistance and increased metastatic risk.

Previous analyses of ZEB1 target genes were mainly focused on EMT-related genes. Repression of epithelial marker genes such as *E-Cadherin*, *Crumbs3*, *PATJ* or the *miR-200* family and activation of mesenchymal markers such as *Vimentin*, *N-Cadherin* or matrix metalloproteinases can sufficiently explain the ZEB1-mediated gain in cell motility and invasiveness^5^. In contrast, ZEB1-related stemness properties and drug resistance were shown to be mainly indirectly achieved through transcriptional repression of miRNAs, such as *miR-200* and *miR-203* (refs 17, 49). Here, we describe a new subset of genes depending on ZEB1 expression, which strongly overlaps with a YAP target gene signature. The importance of YAP/TAZ target genes such as *CTGF*, *CYR61*, *AXL* and others for cancer progression was already demonstrated. CTGF is a secreted extracellular matrix-associated protein and important mediator of the oncogenic properties of YAP. In breast cancer, CTGF is associated with drug resistance^36^,^37^, metastasis^35^ and poor clinical outcome^38^. AXL is an oncogenic transmembrane receptor tyrosine kinase which promotes survival, proliferation, migration and metastasis^50^ and has recently been identified as a YAP target gene^47^,^51^. In MDA-MB231 cells, AXL was shown to be required for the tumorigenic, invasive phenotype and metastatic capacity^52^. Also expression of the YAP-target gene *CYR61* was associated with aggressiveness, therapy resistance and poor clinical outcome in breast cancer^36^,^53^. Notably, a group of known YAP-target genes, which we termed ‘YAP only set' (see Fig. 1d), is not co-activated, but downregulated in the presence of ZEB1. The underlying molecular mechanisms of this differential regulation of YAP target genes by ZEB1 are currently unclear. Strikingly, expression of individual members of the ‘YAP only set' as well as a cluster of the top three genes of this set did not correlate with poor prognosis, but even indicated better therapy response and particularly a reduced metastatic risk. A link to the underlying biology is currently unknown. In summary, the detection of a ‘common ZEB1/YAP target gene set' points to a new mechanism how ZEB1 drives malignant cancer progression, towards invasion, metastasis and therapy resistance.

We wanted to rule out that activation of a common ZEB1/YAP target gene pattern important for tumour progression is a general EMT-associated effect on the Hippo pathway and its effectors, which is indirectly and unspecifically exerted by different EMT-inducers. Upstream regulators of the Hippo pathway include cell polarity factors such as the apical Crumbs and the basal Scribble complex^24^,^39^ and EMT-triggered delocalization or loss of Scribble results in reduced LATS-mediated phosphorylation of YAP/TAZ and subsequent increased protein stability^24^. In addition, the Crumbs complex was shown to interact with YAP/TAZ and leads to its cytoplasmic retention and suppression of TGFβ-induced EMT^39^. We have previously shown that ZEB1 directly represses expression of cell polarity factors including members of the *Crumbs* and *Scribble* family, resulting in a loss of cell polarity^16^,^40^. We ruled out that ZEB1 indirectly stimulates YAP target gene expression by preventing Crumbs- or Scribble-dependent YAP/TAZ degradation or cytoplasmic retention. A specificity of ZEB1 in functional interaction with YAP is further underscored by showing that maintained expression of SNAI2 (Slug) cannot substitute for loss of ZEB1, which also indicates important functional differences between ZEB1 and other EMT-activators, such as SNAI2. In conclusion, indirect EMT-associated and Hippo pathway-mediated regulation of YAP/TAZ activity, protein stability or subcellular localization does not seem to be the primary mechanism of ZEB1 action in this context. Hence, regulation of YAP target genes by ZEB1 cannot simply be ascribed to Hippo-pathway mediated regulation of YAP/TAZ activity, for example, through ZEB1-induced loss of cell polarity.

**Figure 9:**
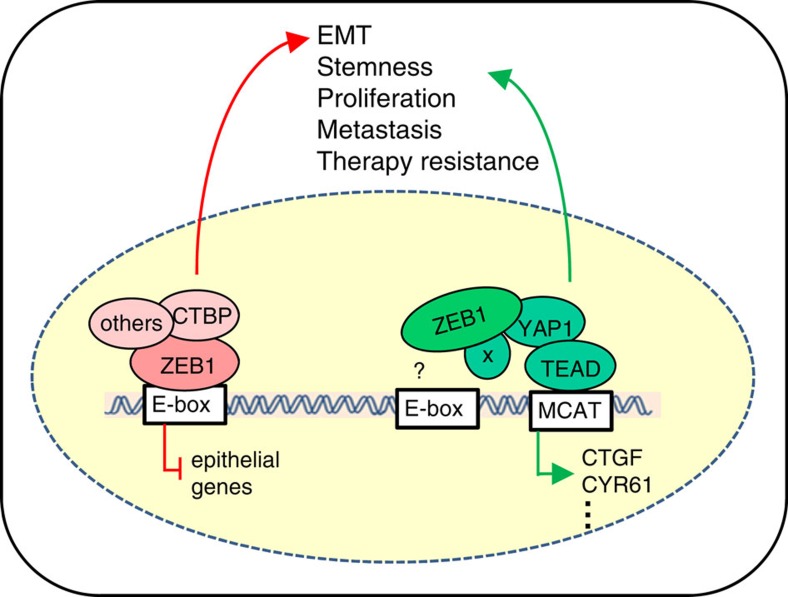
Model of the dual function of ZEB1. ZEB1 is a transcriptional repressor of differentiation-associated genes, thereby inducing EMT. Direct interaction of ZEB1 with YAP (but not with TAZ) switches its function to an activator of a common ZEB1/YAP target gene set, known to stimulate cancer aggressiveness. YAP1 and ZEB1 bind indirectly through MCAT sites of target genes. The role of E-boxes and putative additional binding partners (x) remains to be clarified.

ZEB1 is mainly known as a repressor of epithelial gene expression. Direct transcriptional activation has also been reported for a few target genes and likely involves the recruitment of a different set of co-factors^6^,^54^. Indeed, ZEB1 was found to repress genes when in complex with PC2-CtBP-LSD1-LCoR or the SWI/SNF chromatin-remodelling protein BRG1, whereas formation of a ZEB1-Smad3-p300-P/CAF complex resulted in transcriptional activation^6^. Interestingly, Smad3 can also complex with YAP/TAZ-TEAD and cooperatively induce the expression of *CTGF* downstream of TGFβ signaling in breast cancer cells^7^. Here we show that ZEB1 interacts with YAP, binds to the *CTGF* promoter and co-stimulates activation of a subset of target genes (termed ‘common ZEB1/YAP set') (Fig. 9). Binding of ZEB1 to E-boxes, its well-defined elements for transmitting transcriptional repression, seems to be not important for transcriptional co-stimulation with YAP. Our results indicate that indirect binding to TEAD factors co-activate target genes through MCAT-sites. However a direct DNA-binding of ZEB1 to unknown target sequences cannot be ruled out. Also an interaction with other transcription factors may confer the transcriptional co-activation by ZEB1. Very recently it was shown that AP-1 can co-stimulate transcriptional activation of YAP-target genes. However, AP-1 components do not directly bind to YAP/TAZ, but to TEAD factors and the interaction mainly takes place at target enhancers and not promoters^55^. An involvement of ZEB1 in this interaction is currently not known. YAP interacts with ZEB1 at its N-terminal and C-terminal ends, which were already shown to bind the transcriptional co-activator p300 binding (N-terminal end) and possess a transcriptional activation domain (C-terminal end)^13^,^56^,^57^. We describe that YAP binds to ZEB1 with its central portion containing two WW-domains, already shown to be the binding domain for other transcription factors, such as Runx1,2 and Smad1,7 (refs 29, 58). Notably, we found that TAZ cannot bind ZEB1 with high efficacy. This may be explained by major structural differences between YAP and TAZ, which in contrast to YAP possesses only one WW-domain in the central part^29^,^58^. Thus, our findings demonstrate a significant functional difference of the major Hippo-pathway effectors YAP and TAZ.

We validated the translational and clinical relevance of a functional ZEB1/YAP interaction and its common target gene activation. Survival analysis of breast cancer data sets demonstrated that irrespective of the subtype, tumours with high expression of ZEB1 and ‘common ZEB1/YAP target genes' displayed a significant shorter relapse-free and overall survival. Particularly for hormone receptor-negative cancers (ER−/PR−), which include the most aggressive subtypes^48^, expression of the ‘common ZEB1/YAP target gene set' strongly correlated with poor relapse-free survival, resistance to chemotherapy and high risk for distant metastasis. Notably, expression of the ‘YAP only' target gene set, which is even downregulated in the presence of ZEB1, did not correlate with poor prognosis, but even indicated better therapy response and particularly reduced metastatic risk. We therefore propose that the function of ZEB1 is dominating and determines the clinical outcome. This fits to ambiguous role of Hippo-pathway components on cellular processes described in development and cancer^39^,^59^,^60^,^61^,^62^. For instance, there are also studies, showing that YAP is deleted in breast cancer and that it functions as tumour suppressor^61^. These differences might be linked to the subtype, since an oncogenic YAP function was found in aggressive subtypes, like the basal type, which also express ZEB1. Prospective clinical studies will demonstrate whether the ‘common ZEB1/YAP target gene set' will be a useful tool to predict clinical outcome and therapy response of breast and other cancer types. These results demonstrate a high tumour biological and clinical relevance of a functional interaction between ZEB1 and YAP.

In conclusion, we here describe that, in addition to its established role as transcriptional repressor and EMT-activator, ZEB1 co-activates the expression of a subset of YAP target genes. Thereby, other EMT-activators cannot substitute for ZEB1. ZEB1 directly binds to YAP, but not to its paralogue TAZ, demonstrating an important functional difference between YAP and TAZ. Expression of the identified ‘common ZEB1/YAP target gene set' is a strong predictor of poor clinical outcome in hormone receptor negative breast cancer. We link two pathways with very similar oncogenic and cancer promoting effects^6^,^7^,^25^,^29^ and contribute to the understanding of the pleiotropic roles of ZEB1 and the Hippo-pathway effector YAP in cancer progression and metastasis. Our finding of a direct molecular interaction of ZEB1 and YAP indicates novel strategies to interfere with tumour progression.

## Methods

### Cell culture

Cell lines MDA-MB231, MCF7, MCF10A and HEK293T were purchased from American Type Culture Collection (ATCC). Cell lines were cultured under standard conditions in DMEM (Life Technologies)/10% fetal bovine serum (Gibco) at 37 °C and 5% CO_2_ in a humidified incubator and regularly tested for mycoplasma contamination. Stably transduced MDA-MB231 cell lines (shGFP and shZEB1, ishCTR and ishZEB1) were cultured in 1.0 μg ml^−1^ puromycin. MCF10A cells were cultured in DMEM/F12 (Invitrogen) containing 5% horse serum (Life Technologies), 20 ng ml^−1^ EGF (R&D Systems), 0.5 μg ml^−1^ hydrocortisone (Sigma), 0.1 μg ml^−1^ cholera toxin (Sigma) 10 μg ml^−1^ insulin (Invitrogen) and 10 mM HEPES (Gibco). MDA-MB231 stable knockdown clone for ZEB1 (shZEB1) and control clone (shCTR) were previously described^16^. shYAP MDA-MB231 cells were generated by lentiviral infection with a pGIPZ shYAP knockdown construct (V3LHS_306099, GE Healthcare) or non-silencing control vector and subsequent selection with 2.5 μg ml^−1^ puromycin. MCF10A cells with inducible ZEB1 expression were generated by transfecting MCF10Atet cells with linearized plasmid DNA (pTET-bsr/HAZEB1-IRES-DsRedExpress2) using Amaxa nucleofection. MDA-MB231 Dox-inducible knockdown clones for ZEB1 (ishZEB1) or control (ishCTR) were generated as described^63^. Induction of *ZEB1* expression or knockdown was performed by addition of 1 μg ml^−1^ doxycycline (Dox; Sigma) to the medium for the indicated time. Induction of EMT by TGFβ1 (PeproTech) in MCF10A cells was performed by adding daily 5 ng ml^−1^ TGFβ1 to the medium of MCF10A cultures until cells showed a stable EMT phenotype.

### Plasmids

The CTGF and CYR61-promotor reporter plasmids were generated by PCR amplification from bacmid DNA (RPCIB753I0869Q, RPCIB753E20113Q, Source Bioscience) containing the corresponding human 5′ part of CTGF and CYR61 (CTGF: nucleotides −867 to +55 and −414 to +55; CYR61: −1,100 to +224 and −658 to +224, relative to the transcription start sites (TSSs)) using primers containing SacI/XhoI and SacI/NcoI restriction sites, respectively. The amplicons were inserted into pGL4.10 (E6651, Promega). Mutations of MCAT sites were introduced by sequential QuickchangeII site-directed mutagenesis (Ambion) of the CTGF promotor sequence using complementary primer pairs for MCAT1: 5′-CTTTTTCAGACGGAGtgcgGCTGAGTGTCAAGG-3′ and MCAT2,3: 5′-GGAAGGTGGGGAGtgcgGCGAGtgcgGTCCCTGTTTGTG-3′. The wt and mutant YAP/TAZ reporter constructs were generated by ligating either 5′-GGCCAGTGCCAAGTTGAGACACATTCCACACATTCCACTGCAAGCTTGAGACACATTCCACACATTCCACTGC-3′ or 5′-GGCCAGTGCCAAGTTGAGACACcgcaCACACcgcaCACTGCAAGCTTGAGACACcgcaCACACcgcaCACTGC-3′ synthetic sequences into pGL4.23 (E8411, Promega) using a HindIII linker. pcDNA6-V5-YAP was a gift from Gerd Walz (University Hospital Freiburg, Freiburg, Germany) and the 3XFlag pCMV5-TOPO TAZ WT plasmid was a gift from Jeff Wrana (Addgene plasmid # 24809). V5-tagged YAP mutants that lack the TEAD-binding domain (V5-ΔTEAD-YAP, amino acids 161–504) and transactivation domain (V5-ΔTAD-YAP, amino acids 1–290) and the corresponding wt YAP were cloned into pCIneo (EcoRI) by PCR amplification. For generation of a doxycycline-inducible ZEB1 expression vector, PCR-amplified hemagglutinin (HA)-tagged ZEB1 cDNA was inserted into pMIBerry containing an internal ribosomal entry site (IRES) from the equine meningoencephalitis virus and the red fluorescent protein DsRedExpress2 (ref. 63). The bicistronic HAZEB1-IRES-DsRedExpress2 expression cassette was further used for generation of the pTET-bsr/HAZEB1-IRES-DsRedExpress2 expression plasmid using the NotI site. The pTRIPZ_ishZEB1 plasmid for Dox-inducible knockdown of ZEB1 in MDA-MB231 was generated by cloning of the shRNAmir cassette of pGIPZ ZEB1 (V3LHS_356187, Open Biosystems) into the pTRIPZ vector backbone using XhoI and MluI. pTRIPZ Inducible Lentiviral Non-silencing shRNA Control (Open Biosystems) was used for generation of the MDA-MB231 control cell line.

### Microarrays

Microarray gene expression data for Panc1, MDA-MB231 and HCT116 stable knockdown clones for ZEB1 (shZEB1) and control clones (shCTR) as well as SW480 with transient siRNA mediated knockdown of ZEB1 or control were generated using the Affymetrix HGU-133 Plus 2.0 chip. The microarray data from this publication have been submitted to the ArrayExpress database [ https://www.ebi.ac.uk/arrayexpress/] and assigned the identifiers E-MTAB-3480 (HCT116), E-MTAB-3482 (MDA-MB231), E-MTAB-3483 (Panc1), E-MTAB-3479 (SW480).

### Gene set enrichment analysis and correlation analyses

The expression data set of the cancer cell line encyclopedia panel of breast cancer cell lines was obtained from the NCBI-GEO database (GSE36133; ref. 33) and subtype classification was done according to Prat *et al.*^34^ The human breast tumour microarray data set was obtained from the NCBI-GEO database (GSE18229-GPL1390). A total 186 tumour samples with subtype classification according to Prat *et al.*^34^ were used to analyse the correlation of gene expression and correlations between the gene set and clinical parameters. Correlation of mRNA expression of genes in the gene set in cell lines or human breast tumours were analysed calculating the Pearson correlation coefficient. To evaluate the correlation of the gene set with relapse-free or overall survival, patients were separated into two subgroups, ‘gene set high' or ‘gene set low'. The subgroup classification was performed as described previously^64^. Patients were considered to have a high expression of the gene set if they had average expression values of ≥11 out of the 14 genes in the gene set above the 58th percentile. Vice versa, patients were considered to have a low expression of the gene set if they had average expression values of ≥11 out of the 14 genes in the gene set below the 42th percentile.Kaplan–Meier survival analysis and log-rank test were used to evaluate the statistical significance of survival differences between the two groups. Heat maps were generated using the GENE-E software (Broad Institute). GSEA was performed using the Broad Institute platform (http://www.broadinstitute.org/gsea/index.jsp; Version 2.0.14; refs 65, 66). A total 189 gene sets of the oncogenic signature C6 from the Molecular Signatures database (http://www.broadinstitute.org/gsea/msigdb/genesets.jsp?collection=C6, Broad Institute, MSigDB, Version 4.0) were used for the analysis with default settings and 1,000 gene set permutations. One gene set was filtered out by its size (<15 or >500 genes) and excluded from the analysis. For meta-analysis of published data sets for breast cancer, we used the KM-plotter (http://kmplot.com/analysis)^48^. Patient samples (ER^−^/PR^−^) were selected and grouped for high or low mRNA expression of the selected gene or gene set. All percentiles between the lower and upper quartiles were computed, and the best performing threshold was used as cutoff. The median was calculated over the whole data set. For analyses of gene sets, the ‘multigene classifier' mode was applied.

### siRNA transfection

Transient siRNA-mediated knockdown was performed with Lipofectamine RNAiMAX reagent (Life Technologies) according to the manufacturer's protocol for 72 h. siRNAs were used to a final concentration of 33 nM. For siRNA-mediated knockdown of genes in MCF10A overexpressing ZEB1, cells were first cultured in a flask with or without doxycycline (1 μg ml^−1^) for 3 days. The cells were then plated in six-well plates and transfected the next day. Doxycycline was added again and cells were collected 72 h after transfection. siRNA sequences of silencer select siRNAs (Ambion): hs ZEB1, 5′-GGUAGAUGGUAAUGUAAUAtt-3′ (s229971, Ambion); hs YAP1, 5′-AGAGAUACUUCUUCUUAAAUCAtt-3′ (s20367, Ambion); hs WWTR1, 5′-GUACUUCCUCAAUCACAUAtt-3′ (s24789, Ambion); hs SNAI2, 5′-CAAUAAGACCUAUUCAACUtt-3′ (s13127, Ambion); Silencer Select Negative Control #1 siRNA (4390844, Ambion); hs TAZ, 5′-GUACUUCCUCAAUCACAUAtt-3′ (s24789, Ambion).

### Immunofluorescence and proximity ligation assay

For immunofluorescence, cells were seeded on cover slips. siRNA knockdown or doxycycline treatment was performed after 24 h. The cells were fixed with 4% formaldehyde for 10 min at room temperature (RT), washed twice with wash buffer (0.02% Tween20/PBS) and permeabilized with 0.5% Triton X-100/PBS for 5–10 min at RT. After washing, the cells were incubated with 1.5% BSA/PBS solution (blocking solution) for 30 min at RT. Primary antibodies against ZEB1 (1:200, Santa Cruz, sc-25388), YAP/TAZ (1:400, Santa Cruz, sc-101199) and TAZ (1:200, BD Biosciences 560235) were then incubated in blocking solution at 4 °C overnight. The cells were washed twice and subsequently incubated with Alexa488- and Alexa647-conjugated secondary antibodies for 1 h at RT protected from the light (Life Technologies, A-11034, A-21235, 1:500 in blocking buffer) followed by counterstaining with Hoechst (Molecular Probes). Samples were mounted with ProLong Gold antifade reagent (Life Technologies) and imaged on a confocal microscope (Leica TCS SP2 AOBS). Images were processed using ImageJ software. For proximity ligation assay (PLA), cells were seeded, treated and stained with primary antibodies as for immunofluorescence.The PLA was performed with the Duolink *In Situ* Orange Kit Mouse/Rabbit (Duolink) according to the manufacturer's instructions. Anti-mouse MINUS and anti-rabbit PLUS PLA probes (Duolink) were used. Images were acquired by microscopy (BX-61, Olympus) using a × 40 objective and analysed with the image analysis software Blob Finder V3.2.

### Co-immunoprecipitation

For immunoprecipitation (IP) of proteins from nuclear extracts, V5-YAP and HA-ZEB1 were transiently expressed in MCF7 or 293 cells. The cells were seeded in 150 mm dishes, transfected the next day with 40 μg plasmid per dish in total using FuGENE HD transfection reagent (Promega) according to the manufacturer's instructions and harvested after 72 h. For fractionation of nuclear and cytoplasmic proteins, the cells were first lysed in buffer A (10 mM HEPES pH 7.9, 10 mM KCl, 0.1 mM EDTA, 1 mM DTT, protease inhibitor) for 15 min on ice. Nonidet P40 was added to a final concentration of 0.5% followed by centrifugation of the samples at 13,000*g* for 2 min at 4 °C. Nuclear pellets were washed twice with buffer A, resuspended in buffer B (20 mM HEPES, pH 7.9, 400 mM NaCl, 1 mM EDTA, 1 mM DTT, protease inhibitor) and incubated for 30 min at 4 °C on a shaker. Lysates were cleared by centrifugation at 15,900 RCF for 10 min at 4 °C and equal amounts of protein per sample were subjected to IP. Lysates were incubated with Anti-HA Affinity Matrix (clone 3F10,Roche, 11815016001, 5 μl per 100 ng protein) or Anti-V5-tag mAb-Magnetic beads (MBL, M167-9, 5 μl per 100 ng protein) (MCF7) or anti-V5 antibody (Life Technologies, R-960-25, 1ng per 1 μg protein) and protein A/G UltraLink Resin (Thermo Scientific; 293 cells) for 6 h under rotation at 4 °C. Immunoprecipitates were then washed three times with buffer B and eluted with × 1 NuPAGE LDS Sample buffer (Life Technologies) by incubation at 70 °C for 10 min. Untransfected MDA-MB231 cells were used in same amounts to analyse endogenous YAP-ZEB1 interaction using 250 μg protein and 250 ng anti-YAP (sc-101199, Santa Cruz) antibody.

### *In vitro* protein synthesis and pull-down assays

His-tagged WT YAP, His-tagged WT TAZ, HA-tagged WT ZEB1 and ZEB1 and YAP fragments p1-p3 were synthesized using the RTS100 wheat germ system (5PRIME) according to the manufacturer's instructions. His-tagged YAP and HA-tagged ZEB1 were incubated in pull-down buffer (250 mM sucrose, 10 mM MOPS/KOH pH 7.2, 80 mM KCl, 5 mM MgCl_2_, 5 mM KH_2_PO_4_) for 1 h at 37 °C and 34 RCF. For His-pull-down, 40 μl equilibrated Ni-NTA beads were added and incubated for 1 h at 4 °C and collected by centrifugation at 184 RCF. The Ni-NTA beads were washed 10 times with pull-down buffer containing 40 mM imidazole, and the bound proteins were eluted with pull-down buffer containing 250 mM imidazole. For HA-pull-down, 50 μl equilibrated anti-HA-affinity matrix were added to the mix containing ZEB1 and YAP and transferred to mobicol columns, incubated for 1 h at 4 °C continuously shaking. The HA-matrix was washed five times in pull-down buffer and eluted with laemmli buffer. The proteins were separated by SDS–PAGE and analysed by western blotting.

### Western blotting

For preparation of cell lysates, the cells were washed with ice-cold PBS and lysed in lysis buffer (120 mM NaCl, 50 mM Tris- HCl pH 7.2, 1% Nonidet P40 (v/v), 1 mM EDTA, 6 mM EGTA, 6 mg ml^−1^ sodium pyrophosphate, × 1 protease inhibitor cocktail (Roche) and × 1 phosphatase inhibitor cocktail (Roche)) for 30 min on ice. Lysates were cleared by centrifugation at 15,900 RCF and 4 °C and proteins were separated by SDS–PAGE and transferred to nitrocellulose membrane using the Tetra Cell-Blot (Bio-Rad) with × 1 blotting buffer (20 mM Tris, 150 mM glycine, 20% methanol, pH 8.3). Proteins were detected using rabbit anti-ZEB1 (1:5,000, HPA027524, Sigma), mouse anti-E-cadherin (BD Transduction, 610181; 1:1,000), mouse anti-YAP/TAZ (1:1,000, sc-101199, Santa Cruz), rabbit anti-pYAP (1:1,000, #4911, Cell Signaling), rabbit anti-AXL (1:1,000, #8661, Cell Signaling), goat anti-CTGF (1:500, sc-14939, Santa Cruz), rabbit anti-SNAI2 (1:1,000, #9585, Cell Signaling), mouse anti-HA (1:1,000, MMS-101R, Covance), mouse anti-V5 (1:5,000, R-960-25, Life Technologies) and mouse anti-β-Actin (Sigma, A5441; 1:5,000) or mouse anti-tubulin (1:5,000, T6199, Sigma) as loading control and species-specific secondary HRP-coupled antibodies (Thermo Scientific, #31402, #31430, #31460, 1:12,500). Protein bands were visualized using Amersham ECL Western Blotting Detection Reagents (GE Healthcare) or Amersham ECL Prime Western Blotting Detection Reagents (GE Healthcare) and the ChemiDoc MP system (Bio-Rad).

### Chromatin-immunoprecipitation (ChIP) and ChIP-reChIP

ChIP was performed as previously described^67^, except extra crosslinking with 2 mM DSG for 30 min before the addition of 1% formaldehyde. Chromatin was incubated with anti-ZEB1 (5 μg, Santa Cruz H102, sc-25388X), anti-YAP (5 μg, Santa Cruz, sc-15407X) and normal rabbit IgG control (5 μg Santa Cruz, sc-2345) antibodies overnight and complexes were precipitated by protein A/G Dynabeads (Invitrogen 10002D/10004D, 25 μl each per IP). Precipitates were eluted (0.1 M NaHCO_3_, 1% SDS) and chromatin was de-crosslinked by first incubating 1 h at 37 °C with 250 μg ml^−1^ RNaseA and 500 μg ml^−1^ proteinase K, followed by overnight incubation at 65 °C. After DNA purification the following regions (relative to TSS) of CTGF, CYR61, AXL or SDPR were amplified by quantitative PCR: CTGF: −282 bp to −140 bp (same region as published in Fujii *et al.*^68^); CYR61:−56 bp to −152 bp (same forward primer and region as published in Lai *et al.*^36^; AXL: −1,053 bp to −1,219 bp (same primers as published in Xu *et al.*^47^); SDPR: −761 to −910 bp; HPRT1 was used as negative control.

For ChIP-reChIP experiments, 293 cells were transfected in 15 cm cell culture plates with HA-ZEB1 alone or with HA-ZEB1 and V5-YAP (22.5 and 7.5 μg, respectively) one day after seeding as above^65^,^66^,^67^,^68^. After first ChIP chromatin was eluted from bead-bound antibodies with elution buffer containing 15 mM DTT at 37 °C for 30 min. Pooled chromatin from five individual anti-ZEB1 ChIPs were diluted 1:20 and used in a subsequent anti-V5 ChIP (reChIP) using V5-coupled magnetic beads overnight. After alternate washing steps in 500 mM and 150 mM NaCl containing washing buffer DNA was released as for normal ChIP.

### RNA isolation and quantitative RT–PCR

Total RNA was isolated using the RNeasy Plus Mini Kit (Qiagen) according to the manufacturer's protocol. Reverse transcription of mRNA was performed using the RevertAid First Strand cDNA Synthesis Kit (Thermo Scientific, K1621) according to the manufacturer's instructions. cDNA was amplified using gene-specific primers and Power SYBR Green PCR master mix (Applied Biosystems). Expression values were measured in triplicates on a Roche LightCycler 480 and normalized to human *ACTB* expression. Results are shown as the relative fold expression compared to respective control treatment. All primers used for RT–PCRs are listed in Supplementary Table 1.

### Luciferase reporter assay

Cells were seeded in 24 wells in triplicates and plasmid transfection with FuGENE HD transfection reagent (Promega, E2311) was performed the next day according to the manufacturer's protocol using 270 ng of firefly luciferase reporter vector, 30 ng of pRL-TK *Renilla* luciferase control reporter vector (Promega) and indicated amounts of plasmids encoding YAP1 and/or ZEB1 or the corresponding empty vector controls. Cells were collected after 72 h and luciferase activity was measured using the Dual-Luciferase Reporter Assay system (Promega, E1910) according to the manufacturer's instruction. Expression values of firefly luciferase were normalized to its respective values of *Renilla* luciferase.

### Immunohistochemistry

Immunohistochemistry for ZEB1 on 97 formalin-fixed, paraffin-embedded samples of different histological types of breast cancers from patients who underwent surgery was done as previously described^17^. In brief, sections (4 μm) were deparaffinized, rehydrated and pretreated in a pressure cooker (or in a microwave) in 10 mM citrate buffer pH 6,0. They were then incubated with a polyclonal antiserum against ZEB1, diluted 1:800 (Sigma, #HPA027524) at 4 °C overnight. Slides were washed three times with TBS/0,05% Tween20 and developed with the EnVision-System (DAKO, #K4003) according to the manufacturer's protocol. Finally they were counterstained with Mayer's haematoxylin, diluted 1:10 (Merck; #1.09249.0500) for 60–90 s. For detection of CTGF and YAP1 expression, samples were stained with rabbit anti CTGF (Abcam ab6992, diluted 1:150) and rabbit anti YAP1 (Proteintech #13584-1-AP, diluted 1:400). Sections were masked and analysed independently by two researchers. Samples were retrieved from local archives and usage was approved by the Ethics Committee of the University of Erlangen-Nuremberg (no. 374–14 Bc). Informed consent was provided by the patients when required.

### Statistical analyses

All statistical and correlation analyses were performed using GraphPad Prism 6 software (GraphPad Software, Inc.). Each experiment was repeated three times or more and all the data are expressed as mean±s.e.m. Unpaired two-tailed Student's *t*-test was applied to compare two groups of independent samples and normal distribution was assumed. Comparison between three or more groups was performed using two-way analysis of variance with Bonferroni *post hoc t*-test multiple comparisons test. Significant enrichment of specific antibodies compared to IgG control in ChIP assays was evaluated by two-sided Mann–Whitney *U*-test. Correlation of mRNA-mRNA pairs of the gene set in cell lines or human breast tumours were analysed calculating the Pearson correlation coefficient. The log-rank test was used to compare Kaplan–Meier survival curves. Statistical significance is presented as follows: *P≤0.05; **P≤0.01; ***P≤0.001; ****P≤0.0001; NS, not significant.

## Additional information

**Accession codes:** The microarray data have been deposited in the ArrayExpress database under accession codes E-MTAB-3480 (HCT116), E-MTAB-3482 (MDA-MB231), E-MTAB-3483 (Panc1), E-MTAB-3479 (SW480).

**How to cite this article:** Lehmann, W. *et al.* ZEB1 turns into a transcriptional activator by interacting with YAP1 in aggressive cancer types. *Nat. Commun.* 7:10498 doi: 10.1038/ncomms10498 (2016).

## Supplementary Material

Supplementary Information Supplementary Figures 1-6, Supplementary Table 1 and Supplementary References.
