# Supplementary material for: ZEB1 turns into a transcriptional activator by interacting with YAP1 in aggressive cancer types
Source: Nat Commun. 2016 Feb 15;7:10498. doi: 10.1038/ncomms10498 (PMC4756710; doi:10.1038/ncomms10498)
Supplement: Supplementary Information — Supplementary Figures 1-6, Supplementary Table 1 and Supplementary References. [file ncomms10498-s1.pdf]

a

	NAME	SIZE	ES	NES	NOM p-val	FDR q-val
1	BMI1_DN_MEL18_DN.V1_UP	131	0.65	2.71	< 0.0001	0.000
2	MEL18_DN.V1_UP	130	0.58	2.44	< 0.0001	0.000
3	CORDENONSI_YAP_CONSERVED_SIGNATURE	54	0.63	2.23	< 0.0001	0.000
4	HINATA_NFKB_IMMU_INF	17	0.81	2.22	< 0.0001	0.000
5	BMI1_DN.V1_UP	142	0.49	2.06	< 0.0001	0.000

b

CORDENONSI_YAP_CONSERVED_SIGNATURE		
CTGF	AMOTL2	BICC1
DLC1	DDAH1	ITGB5
TGFB2	SERPINE1	STMN1
CYR61	MARCKS	TNNT2
ANKRD1	FLNA	DUT
FSTL1	THBS1	ETV5
ITGB2	TOP2A	PDLIM2
LHFP	MDFIC	GGH
CRIM1	PMP22	SH2D4A
SLIT2	GLS	TSPAN3
DUSP1	GADD45B	COL4A3
CDKN2C	ECT2	BIRC5
DAB2	NDRG1	TK1
GAS6	CENPF	TNS1
SCHIP1	SHCBP1	HMMR
SDPR	HEXB	PHGDH
TGM2	CDC20	FSCN1
AXL	EMP2	FGF2

c

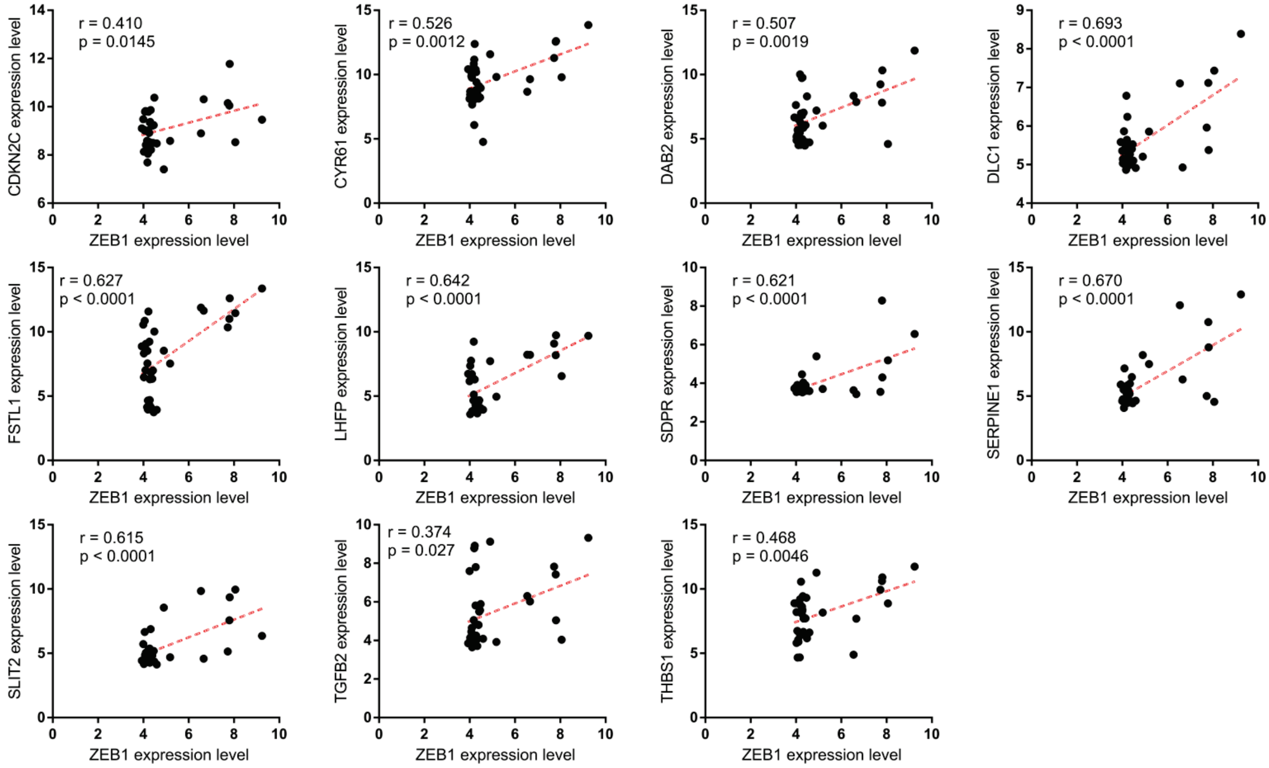

Supplementary Figure 1. Correlated expression of ZEB1 and YAP target genes

(a) The table lists the top 5 of 189 tested gene sets showing the strongest enrichment in the shCTR phenotype, indicating downregulation of the included genes upon stable knockdown of ZEB1 in MDA-MB231. **(b)** The YAP/TAZ signature as defined by Cordenonsi et al. (1) consisting of 54 conserved YAP target genes. **(c)** Analysis of a panel of breast cancer cell lines from the cancer cell line encyclopedia (CCLE) demonstrates significant correlations between mRNA expression of ZEB1 and YAP target genes. For statistical analysis the Pearson correlation coefficient was used.

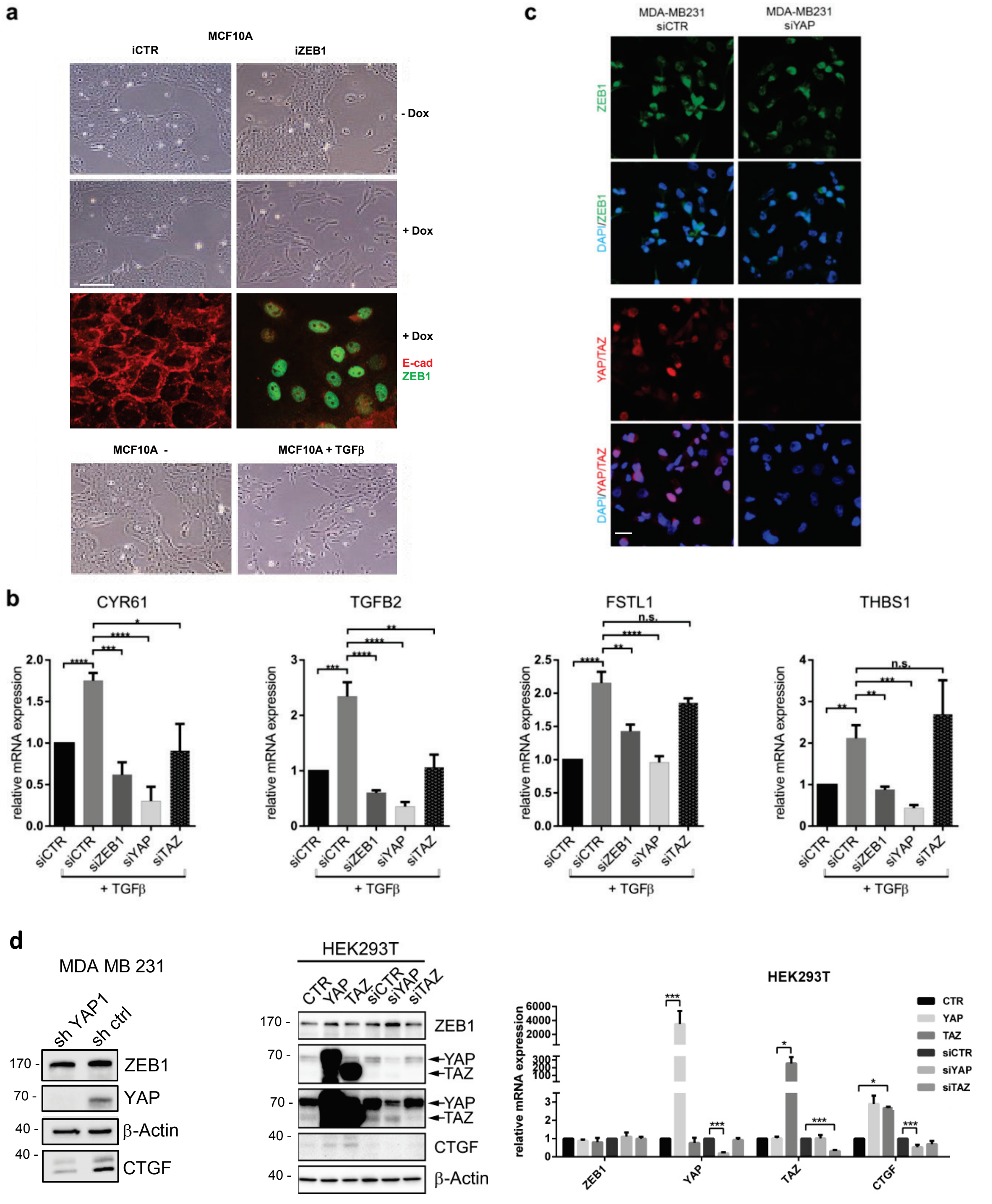

Supplementary Figure 2. ZEB1 regulates YAP target genes

(a) Phase contrast images of MCF10A cells showing phenotypic changes after induction of ZEB1 expression by doxycycline for 6 days and by longterm treatment with TGFβ. Immunofluorescence staining shows downregulation of E-cadherin after induction of ZEB1. Scale bars 40 µm for phase contrast and 20 µm immunofluorescence images. **(b)** Depletion of ZEB1, YAP or TAZ reverts TGFβ-induced expression of YAP target genes in MCF10A cells. siRNA-mediated knockdown in untreated or TGFβ-treated MCF10 cells was performed for 72 h and cells were analyzed by qRT-PCR. n= 6 **(c)** Immunofluorescence of MDA-MB231 cells showing that siRNA-mediated knockdown of YAP does not affect ZEB1 subcellular localization. Nuclear counterstaining by DAPI. Scale bar 20 µm. **(d)** Modulation of YAP or TAZ does not affect the expression of ZEB1 on protein or mRNA level, as shown by RNAi-RNA mediated depletion of endogenous YAP and TAZ or overexpression of YAP and TAZ in HEK293 cells. n=3. For (b) and (c): mean ± SEM. *p=0.01-0.05, **p= 0.001-0.01, ***p= 0.001- 0.0001, ****p<0.0001, n.s. = not significant, unpaired two-tailed Student's t-test.

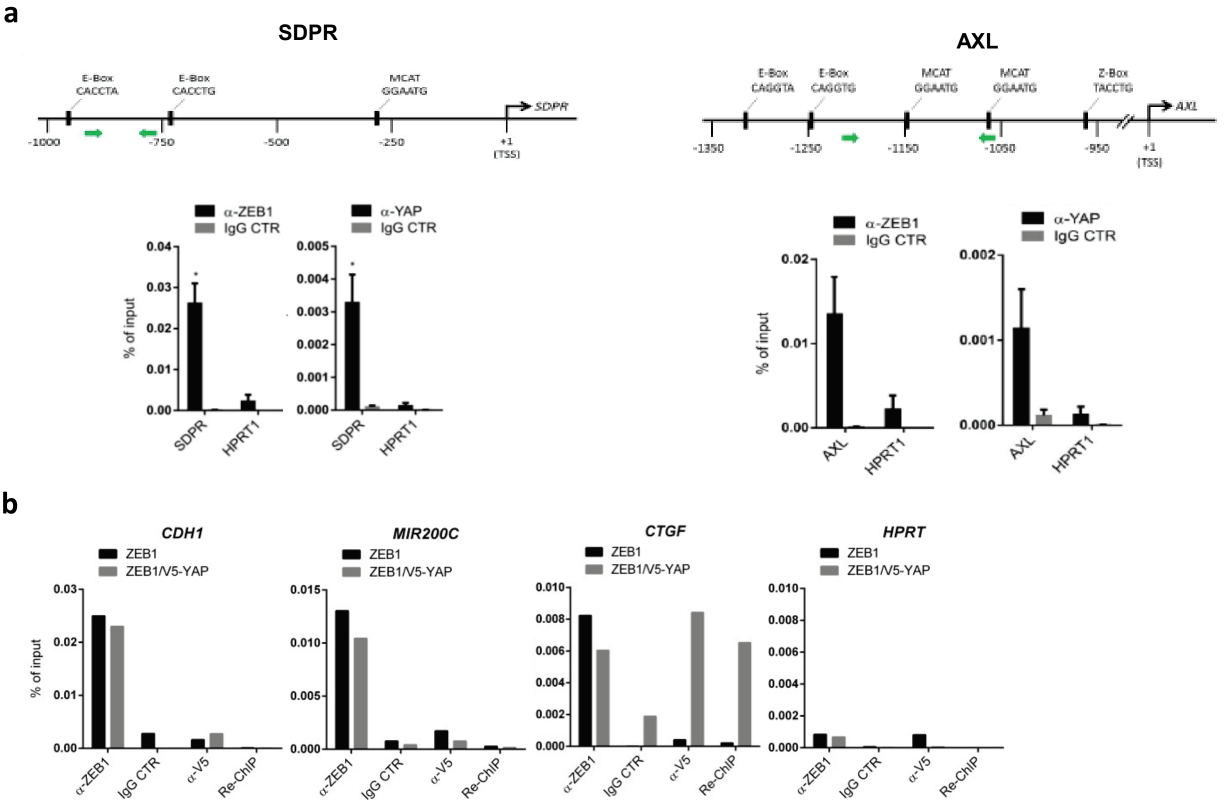

Supplementary Figure 3. ZEB1 functionally interacts with YAP

(a) Schematic representation of the promoter region of human *CYR61* and *Axl* genes. The potential ZEB1 (E-boxes) and YAP/TEAD (MCAT) binding sites and the regions amplified after chromatin immunoprecipitation (ChIP) (green arrows represent primer pairs) are depicted. Numbers indicate positions in bp on chromosomal DNA relative to the transcription start site (TSS). qPCR analysis of ChIP samples using antibodies to precipitate endogenously expressed ZEB1 and YAP in MDA-MB231 cells shows direct binding of ZEB1 and YAP to the promoter of *CYR61 and Axl*. *HPRT1* is used as negative control. n=3, data are expressed as mean ± SEM. *p=0.01-0.05, nonparametric Mann-Whitney-U test. **(b)** ChIP-re-ChIP: qPCR analyses for the depicted four genes after ChIPs with the indicated antibodies after overexpression of ZEB1 with and without V5-tagged YAP in HEK293 cells (first ChIP using indicated antibodies and re-ChIP against V5 after anti-ZEB1 ChIP). Note that re-ChIP for V5-tagged YAP after initial anti-ZEB1 ChIP works only for the CTGF locus, but not for the *CDH1* and *miR-200c* loci, two genes repressed and bound only by ZEB1. HPRT locus was used as negative control.

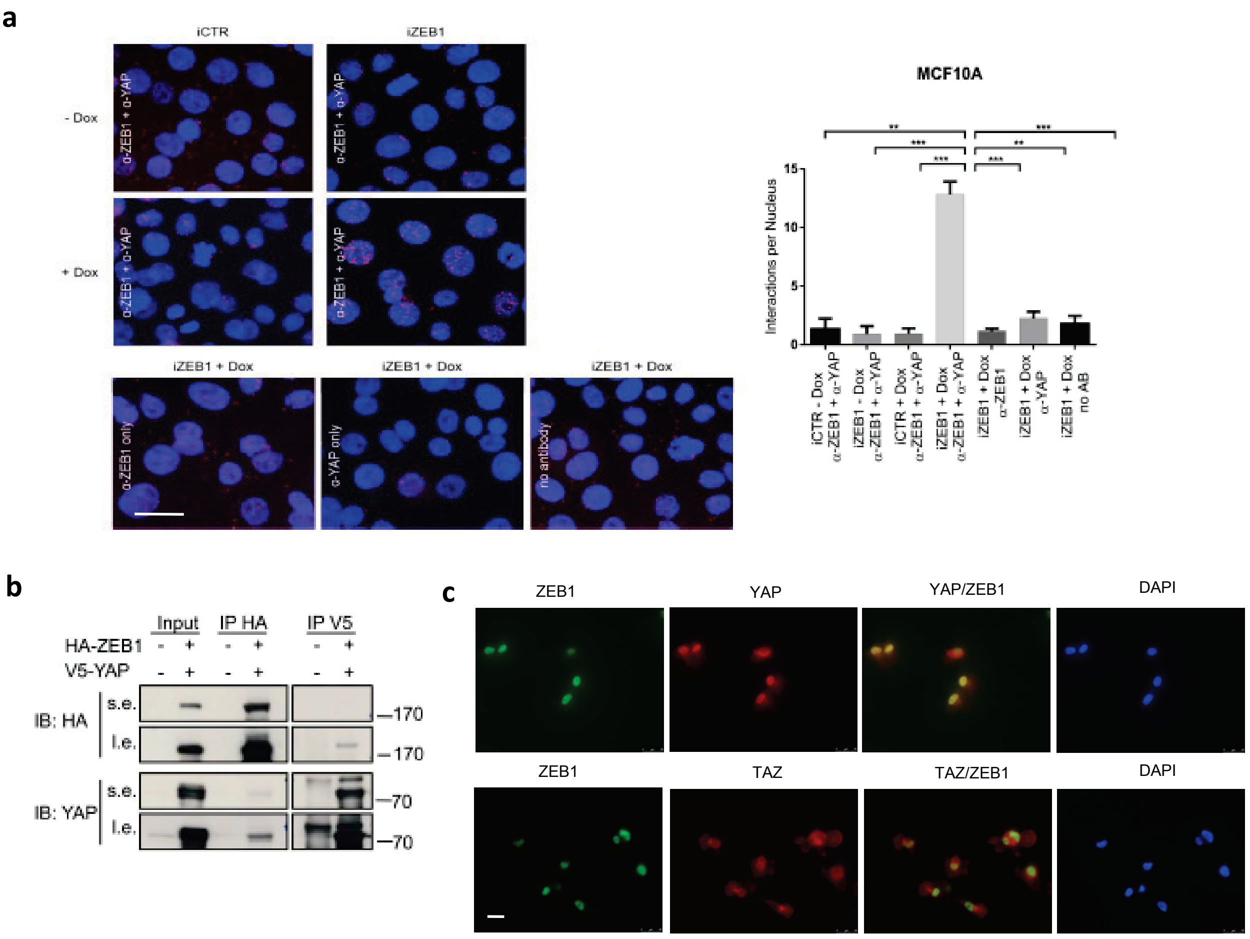

Supplementary Figure 4. ZEB1 directly interacts with YAP

(a) *In situ* proximity ligation assay (PLA) shows sites of interaction between ZEB1 and YAP/TAZ in the nucleus of ZEB1-expressing MCF10A cells indicated by red fluorescent spots. Expression of ZEB1 in MCF10A cells was induced by addition of doxycycline. Uninduced cells or cells carrying the control plasmid or incubation without antibody or either ZEB1 or YAP antibody alone were used as controls. Representative microscopic images are shown and five images from each condition were quantified, counting the number of interactions per nucleus. n= 3, mean ± SEM. ****$p<0.0001$, unpaired two-tailed Student's t-test. Scale bar 10 µm. **(b)** Co-immunoprecipitation of ectopically expressed HA-tagged ZEB1 and V5-tagged YAP from nuclear extracts of HEK293 cells reveals interaction between ZEB1 and YAP proteins on western blot. IB: immunoblot; s.e: short exposure, l.e.: long exposure. **(c)** Immunofluorescence stainings (control for PLAs) showing nuclear expression of ZEB1, YAP and TAZ in MDA-MB231; scale bar 10 µm.

a

‘common ZEB1/YAP target genes‘

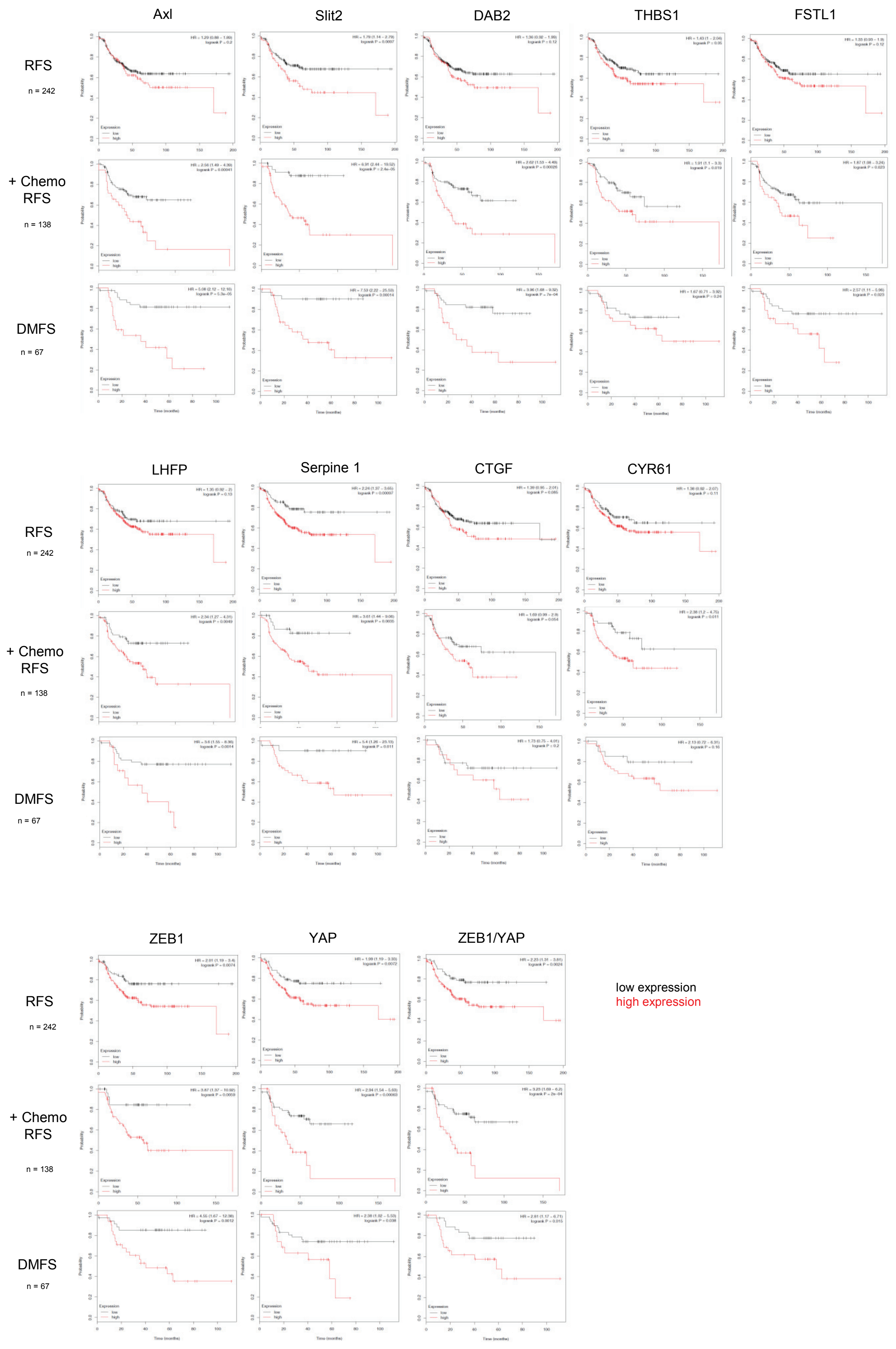

b

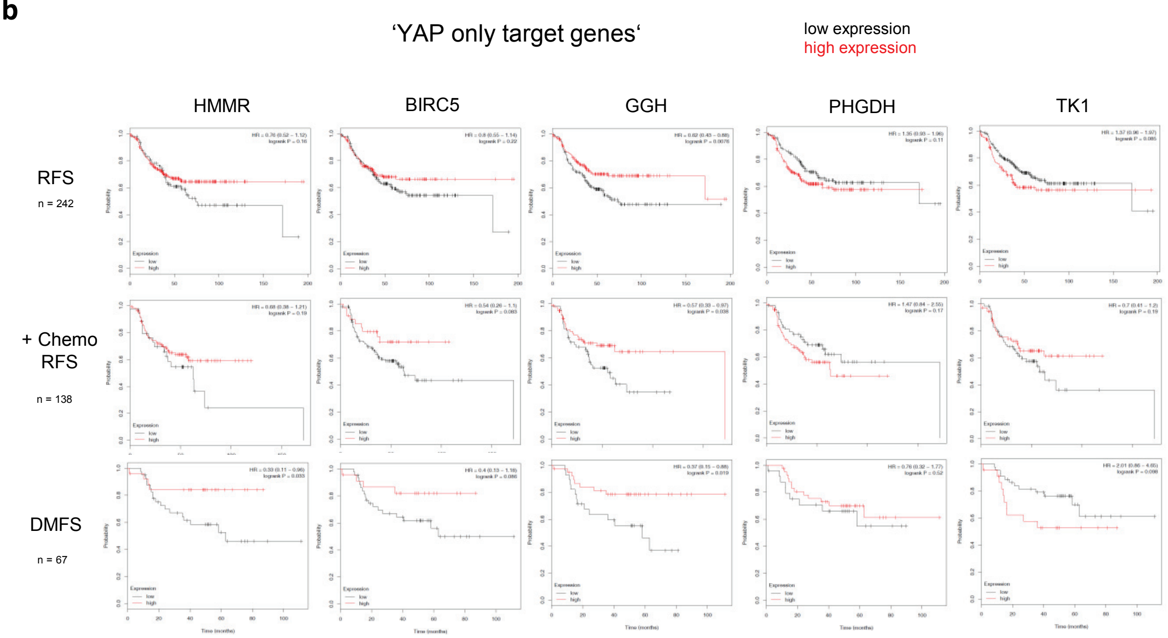

Supplementary Figure 5. Clinical relevance of common ZEB1/YAP target genes

(a) Kaplan-Meier plots from meta-analyses showing relapse-free survival (RFS), relapse-free survival after adjuvant chemotherapy and distant metastasis-free survival (DMFS) of ER^-/PR^- breast cancers based on expression of ZEB1, YAP and the indicated genes of the 'common ZEB1/YAP target gene set'. **(b)** Kaplan-Meier plots from meta-analyses showing relapse-free survival (RFS), relapse-free survival after adjuvant chemotherapy and distant metastasis-free survival (DMFS) of ER^-/PR^- breast cancers based on expression of the indicated genes of the 'YAP only target gene set'. Note the inverse behavior compared to genes of the 'common ZEB1/YAP target gene set' in (a). Log-rank test.

Fig. 2 a

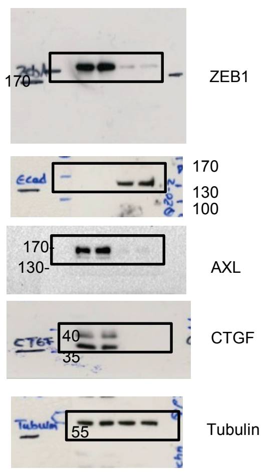

Fig. 2 b

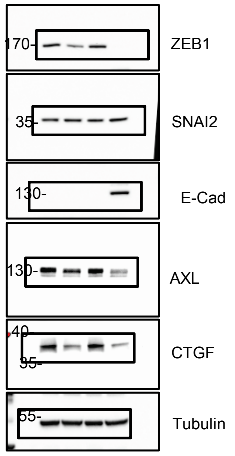

Fig. 2 c

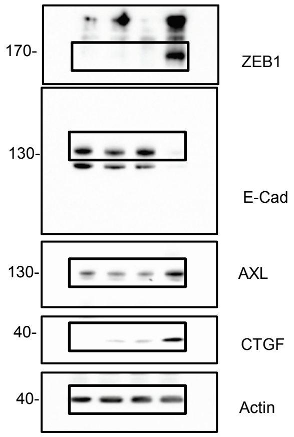

Fig. 2d

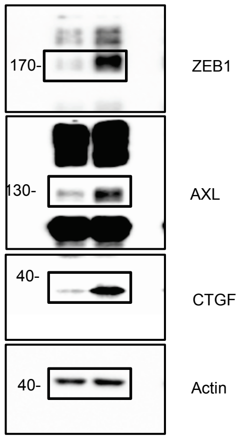

Fig. 3 a

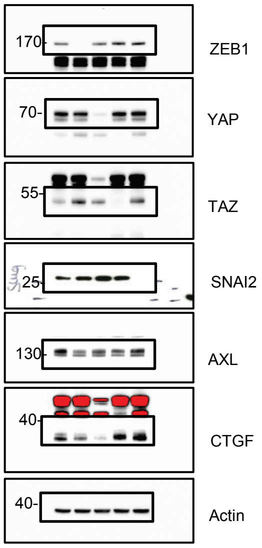

Fig. 3 b

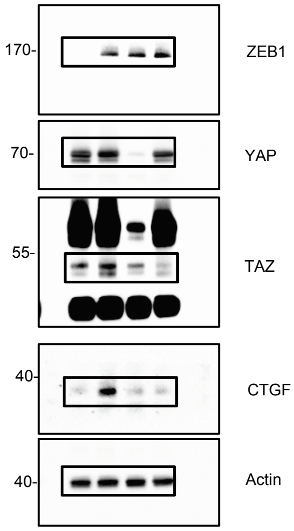

Fig. 3 c

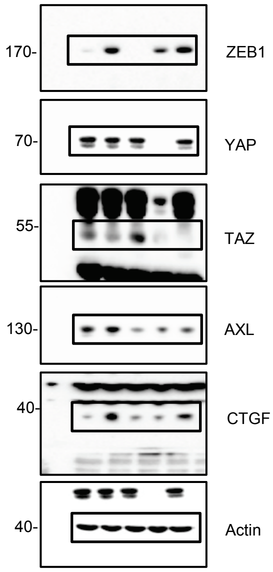

Fig. 4 a

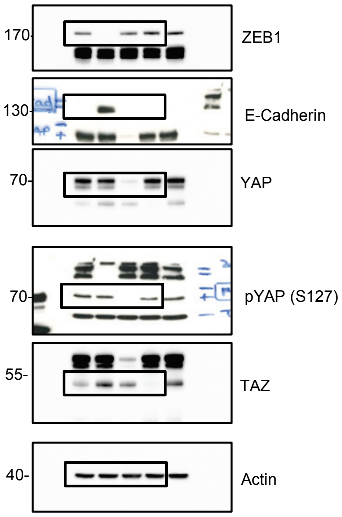

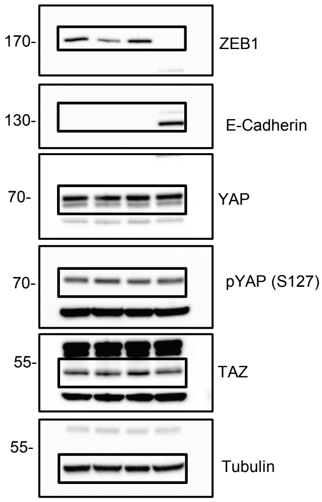

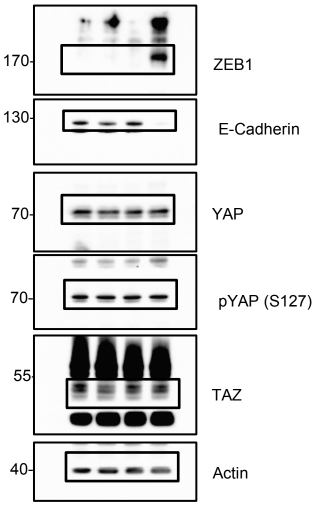

Supplementary Figure 6. Source data, western blots, 1

Fig. 6b

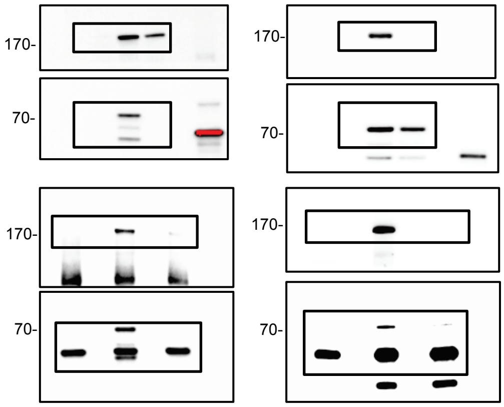

Fig. 6c

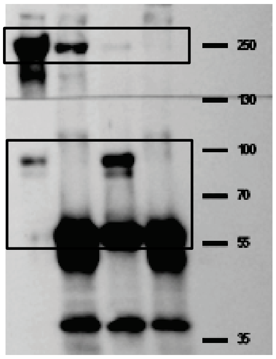

Fig. 6d

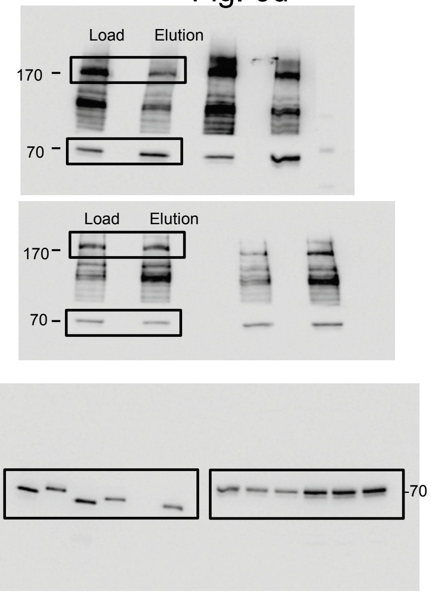

Fig. 6e

Fig. 6g

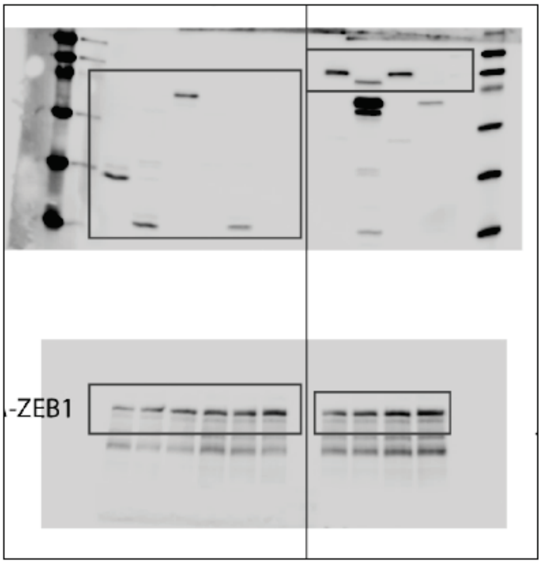

Suppl. Fig. 2d

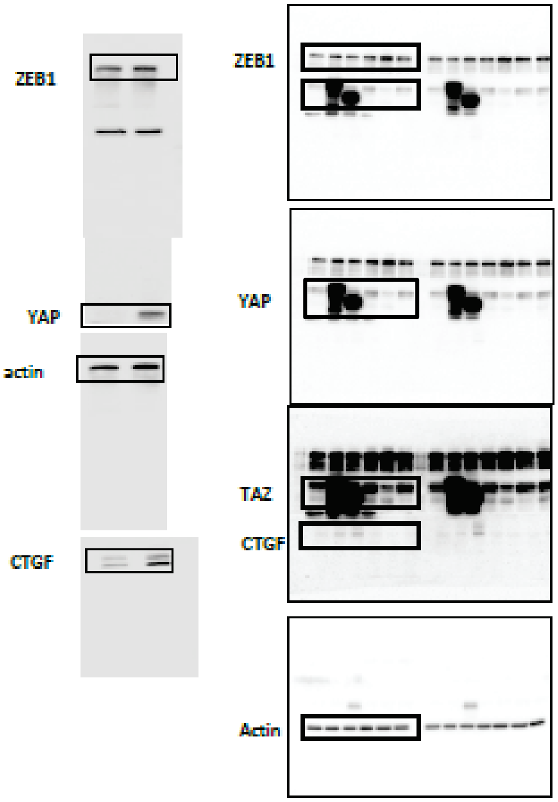

Suppl. Fig. 4b

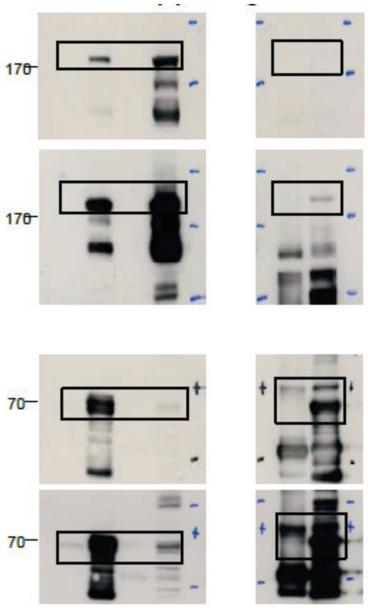

Supplementary Figure 6. Source data, western blots, 2

Supplementary Table 1

Oligonucleotides

The following primers for q-RT-PCR were used:

hs ZEB1, s 5’-AAGAATTCACAGTGGAGAGAAGCCA-3’,
as 5’-CGTTTCTTGCAGTTTGGGCATT-3’;
hs ACTB, s 5’-GCCCTGAGGCACTCTTCCA-3’, as 5’-TTGCGGATGTCCACGTCA-3’;
hs YAP1, s 5’ ATCCCAGCACAGCAAATTCT-3’, as 5’-TGGATTTTGAGTCCCACCAT-3’;
hs WWTR1, s 5’GGCTGGGAGATGACCTTCAC-3’, as 5’-CTGAGTGGGGTGGTTCTGCT-3’;
hs SNAI2, s 5’TGGTTGCTTCAAGGACACAT-3’, as 5’-GTTGCAGTGAGGGCAAGAA-3’;
hs CDKN2C, s 5’GACTATCCCTTCGGCGAGA-3’, as 5’-AAGGCTCGGCCATTCTTTAG-3’;
hs SDPR, s 5’CTCCGACGCAACCATTTC-3’, as 5’-AAACGGGCTGTTTCACAAA-3’;
hs CTGF, s 5’CCTGCAGGCTAGAGAAGCAG-3’, as 5’-TGGAGATTTTGGGAGTACGG-3’;
hs AXL, s 5’CGTAACCTCCACCTGGTCTC-3’, as 5’-TCCCATCGTCTGACAGCA-3’;
hs SERPINE1, s 5’AAGGCACCTCTGAGAACTTCA-3’, as 5’-CCCAGGACTAGGCAGGTG-3’;
hs DAB2, s 5’CCCACCTCCACAAAGTACCA-3’, as 5’-CAAGCAAGTCATTGGCTGAA-3’;
hs FSTL1, s 5’GCCATCAATATTACAACGTATCCA-3’, as 5’-TCAATGAGAGCATCAACACAGA-3’;
hs CYR61, s 5’AAGAAACCCGGATTTGTGAG-3’, as 5’-GCTGCATTTCTTGCCCTTT-3’;
hs DLC1, s 5’TTCCATCTCAACACCCTGAA-3’, as 5’-TGGTTTGCCCAAACTTTGTT-3’;
hs TGFB2, s 5’CCAAAGGGTACAATGCCAAC-3’, as 5’-CAGATGCTTCTGGATTTATGGTATT-3’;
hs LHFP, s 5’TCCAGGACAGTGGGAAGAGT-3’, as 5’-GCCAGCACCAATCAACAAG-3’;
hs THBS1, s 5’CAATGCCACAGTTCCTGATG-3’, as 5’-TGGAGACCAGCCATCGTC-3’;
hs SLIT2, s 5’CCCCAAAAGTTTATTTGAAGGAC-3’, as 5’-CCCGAAGGCAGTTTATCTTG-3’

Supplementary References

(1) Cordenonsi, M., *et al.* The Hippo Transducer TAZ Confers Cancer Stem Cell-Related Traits on Breast Cancer Cells. *Cell* **147**, 759-772 (2011).
